# EREG is the core onco-immunological biomarker of cuproptosis and mediates the cross-talk between VEGF and CD99 signaling in glioblastoma

**DOI:** 10.1186/s12967-023-03883-4

**Published:** 2023-01-16

**Authors:** Yujie Zhou, Dongdong Xiao, Xiaobing Jiang, Chuansheng Nie

**Affiliations:** Department of Neurosurgery, Union Hospital, Tongji Medical College, Huazhong University of Science and Technology, 1277 Jiefang Avenue, Wuhan, 430022 Hubei China

**Keywords:** Cuproptosis, Bulk RNA-seq analysis, Single cell RNA-seq, Immunohistochemistry, Transwell assays, Flow cytometry cell cycle, VEGFA, CD99, Immune microenvironment

## Abstract

**Background:**

Glioma is the most prevalent primary tumor of the central nervous system. Glioblastoma multiforme (GBM) is the most malignant form of glioma with an extremely poor prognosis. A novel, regulated cell death form of copper-induced cell death called “cuproptosis” provides a new prospect for cancer treatment by regulating cuproptosis.

**Methods:**

Data from bulk RNA sequencing (RNA-seq) analysis (The Cancer Genome Atlas cohort and Chinese Glioma Genome Atlas cohort) and single cell RNA-seq (scRNA-seq) analysis were integrated to reveal their relationships. A scoring system was constructed according to the cuproptosis-related gene expression, and core genes were experimentally verified using real-time quantitative reverse transcription polymerase chain reaction (qRT-PCR), Western blot (WB), immunohistochemistry (IHC), and immunofluorescence (IF). Moreover, cell counting kit-8 (CCK8), colony formation, 5-ethynyl-2’-deoxyuridine (EdU) incorporation, transwell, and flow cytometry cell cycle were performed to evaluate cell proliferation, invasion, and migration.

**Results:**

The Cuproptosis Activation Scoring (CuAS) Model has stable and independent prognostic efficacy, as verified by two CGGA datasets. Epiregulin (EREG), the gene of the model has the most contributions in the principal component analysis (PCA), is an onco-immunological gene that can affect immunity by influencing the expression of programmed death-ligand 1 (PD-L1) and mediate the process of cuproptosis by influencing the expression of ferredoxin 1 (FDX1). Single cell transcriptome analysis revealed that high CuAS GBM cells are found in vascular endothelial growth factor A (VEGFA) + malignant cells. Oligodendrocyte transcription factor 1 (OLIG1) + malignant is the original clone, and VEGF and CD99 are the differential pathways of specific cell communication between the high and low CuAS groups. This was also demonstrated by immunofluorescence in the tissue sections. Furthermore, CuAS has therapeutic potential for immunotherapy, and we predict that many drugs (methotrexate, NU7441, KU -0063794, GDC-0941, cabozantinib, and NVP-BEZ235) may be used in patients with high CuAS.

**Conclusion:**

EREG is the core onco-immunological biomarker of CuAS and modulates the cross-talk between VEGF and CD99 signaling in glioblastoma, and CuAS may provide support for immunotherapy and chemotherapy.

**Supplementary Information:**

The online version contains supplementary material available at 10.1186/s12967-023-03883-4.

## Background

Glioma is the most prevalent primary tumor of the central nervous system [1]. It is classified into grades I to IV by the World Health Organization based on its malignant features, wherein grades I, II, and III are low-grade glioma and grade IV is high-grade glioma, also known as glioblastoma multiforme (GBM) [1, 2]. GBM is the most malignant form of glioma with an extremely poor prognosis. Despite considerable advances in the development of treatments, including surgical resection, radiotherapy, and chemotherapy, little progress toward prolonged survival and better prognosis has been achieved over the last few decades [3]. The modest median overall survival (OS) time in GBM is approximately 14 months, and only 5% to 6.8% of patients with GBM survive 5 years after diagnosis [1–4]. Multiple clinical trials, including those on immunotherapy, have been conducted for patients with GBM; however, the results did not conclude the expected results [1–4]. In our previous study, we generated a ferroptosis-related prognostic risk score model to predict the clinical significance and immunogenic characteristics of GBM [5]. However, the biomarkers and predictors for patient outcome and the immunotherapy response of GBMs have not been fully elucidated, and existing predictive models are far from satisfactory.

Beyond classical apoptosis, several forms of regulated cell death (RCD) have been identified, such as ferroptosis, necroptosis and pyroptosis [6]. These RCD subroutines differ in the initiating stimuli, intermediate activation events, and end effectors. Various heavy metals are essential micronutrients; however, the insufficiency or excessive abundance of metals can trigger cell death, which can induce RCD through different subroutines. For example, ferroptosis has been defined as an iron-dependent form of oxidative cell death caused by unrestricted lipid peroxidation [7]. A novel RCD form of copper-induced cell death called “cuproptosis” was proposed by Tsvetkov et al. [8], which is gaining attention in the field. Cuproptosis differs from oxidative stress-related cell death (e.g., apoptosis, ferroptosis, and necroptosis). In contrast, mitochondrial stress, especially the aggregation of lipoylated proteins and destabilization of Fe-S cluster proteins, results in proteotoxic stress and ultimately cell death. Hence, it may provide a new prospective for cancer treatment by regulating cuproptosis.

## Methods

### Data preparation

The omics data of the GBM samples from the TCGA database, including mRNA expression, Single Nucleotide Variant (SNV), copy number variation (CNA), and clinical information, were downloaded from UCSC Xena (https://xenabrowser.net/). The mutation, CNA, and intracomatous heterogeneity of samples were derived from previous studies [9]. The sample set from the CGGA (http://www.cgga.org.cn/) database was used as an independent validation set, involving 139 (CGGA1-mRNAseq325) and 124 (CGGA2-mRNA-Array301) GBM samples. Data for scRNA-seq were downloaded from the GEO (https://www.ncbi.nlm.nih.gov/geo/) database (GSE173278). Clinical characteristics of the three data sets were summarized (see Additional file 1: Table S1).

### Cuproptosis activation scoring model

Ten cuproptosis characteristic genes (FDX1, LIAS, LIPTI, DLD, DLAT, PDHA1, PDHB, MTF1, GLS, and CDKN2A) were obtained from previous studies [8]. Based on these characteristic genes, consistent clustering analysis was used to identify two sample clusters in the TCGA-GBM sample. The gene expression count data of two sample clusters were calculated based on R packet DESeq2 to identify differentially expressed genes (DEGs) [10]. Candidate prognostic genes were identified from differentially expressed genes based on a univariate Cox regression analysis, and redundant factors were further filtered using LASSO Cox analysis. Based on the contribution of the prognostic genes to principal components 1 and 2, CuAS was defined as:

$$cuproptosis activation score (CuAS) = {Gene}_{HR > 1}* (PC1 + PC2)- {Gene}_{HR < 1} * (PC1 + PC2)$$

where HR (hazard ratio) is derived from the Cox analysis. The CuAS model in the validation cohort was reproduced with a similar formula. The construction of the cluster and CuAS models is illustrated in a schematic diagram (Fig. 14).

### Biofunction prediction

The GO/KEGG enrichment analysis based on GSVA calculated the GO/KEGG signature activity scores in each sample, and the significant differences in the activity scores between the sample groups were compared. In addition, gene set enrichment analysis (GSEA) was used to calculate the differential expression of genes between the high and low CuAS groups, and the enrichment significance was calculated.

### Overall survival outcome prediction

Samples were grouped into the high or low CuAS groups according to the median value of the CuAS. The OS difference between the high and low CuAS groups was predicted with the Kaplan–Meier algorithm. The receiver operating characteristic (ROC) curve and area under the curve (AUC) were generated to compare the prognostic ability within the different models.

### GBM immune landscape

The immune and stroma scores and the tumor purity of the tumor samples were calculated based on the ESTIMATE algorithm [11]. The cell composition of the tumor microenvironment (cellular infiltration) was calculated based on the CIBERSORT [12] and xCell algorithms [13] and GSVA score of the 28 immune cell signature genes, respectively [14].

### Tissue sampling from glioma patients

Fresh GBM tissues from histologically confirmed cases were obtained from the Union Hospital, Tongji Medical College, Huazhong University of Science and Technology. The study was approved by the ethics committee of the Union Hospital, Tongji Medical College, Huazhong University of Science and Technology.

### Cell culture, real-time polymerase chain reaction, and immunohistochemistry

The normal human astrocyte cell line HA1800 and human glioma tumor cell lines U87, U251, LN229 and A172 were purchased from the Cell Bank of the Chinese Academy of Sciences. The STR identification reports of the cancer cell lines are presented in Additional materials (see Additional materials-cell lines STR identification), and we also used CCLA, an excellent cell line identification database, for secondary identification to ensure no cross-contamination of cell lines [15]. The cells were cultured in Dulbecco’s Modified Eagle’s Medium (DMEM) (Gibco) containing 10% heat-inactivated fetal bovine serum and 1% penicillin/streptomycin. qRT-PCR was conducted to compare the gene expression in 20 tumor samples in adjacent normal tissue. qRT-PCR was performed in triplicate using samples derived from three independent experiments. Formalin-fixed, paraffin-embedded GBM tissues were used for IHC staining. The primers’ sequence are provided (see Additional file 1: Table S3).

### Lentivirus infection assay

The assay complies with the protocol described in a previous article [16]. Short hairpin RNA (shRNA) against EREG (shEREG) and a negative control shRNA (sh NC) were designed and synthesized by GeneCreate (Wuhan, China). The lentivirus, pLent-shEREG-Flag-Puro or its negative control (NC) pLent-Flag-Puro (GeneCreate) was used to infect GBM cells with enhanced infection solution (GeneCreate) according to the manufacturer’s protocol. Seventy-two hours after the cells were infected with lentivirus, 2 μg/mL puromycin was added to kill the cells that had not been transfected. shRNA sequences are provided (see Additional file 1: Table S2).

### Cell counting kit-8 assay

U87 and U251 cells were assessed with the CCK-8 (Biosharp, China) reagent according to the manufacturer’s instructions. Cells were inoculated on 96-well plates at a density of 2000 cells per well with 100 μL of medium. CCK8 solution (10 μL) was added to each well every 24 h for a total of 96 h, and the cells were further incubated at 37 °C for 1 h. The absorbance of each well was measured at 450 nm with a spectrophotometer.

### Colony formation assay

U87 and U251 cells were prepared into a single cell suspension and seeded into a six-well plate (200 cells/well) for a two-week incubation to form colonies. After staining with 0.01% crystal violet (Sigma), the colonies were subjected to microscopic examination. The rate of colony formation was calculated.

### Cell invasion and migration assays

After starving the cells for 6–8 h in serum-free DMEM, a total of 1 × 10^4^ cells were seeded in the upper chamber with 200 μl of serum-free medium for the migration assay. In addition, 2 × 10^4^ cells were added into Matrigel‑coated upper transwell chambers for the invasion assay. The lower chambers were filled with DMEM containing 10% FBS. After incubation at 37 °C for 24 h, cells on the lower surface of the membrane were fixed in 100% methanol and stained with 0.1% crystal violet dye for 20 min at room temperature. Finally, after washing with phosphate-buffered saline, the cells were imaged in five randomly selected fields under a light microscope (Olympus Corporation) at × 100 magnification.

### 5-ethynyl-2’-deoxyuridine (EdU) incorporation assay

According to the manufacturer’s instructions, the EdU Kit (Roche, Mannheim, Germany) was utilized to monitor the proliferation of transfected cells. A Zeiss Axiophot Photomicroscope (Carl Zeiss, Oberkochen, Germany) was used to capture representative images.

### Compound

Elesclomol (STA-4783) was obtained from MedChemExpress (MCE). CuCl_2_ (Copper (II) chloride, 97%, 222011), and FeCl_3_ (reagent grade, 97%, 157740) were obtained from Sigma-Aldrich.

### Western blotting

Proteins from tissues and cells were extracted using radioimmunoprecipitation assay (RIPA) (strong) buffer (Beyotime, Shanghai, China) containing protease inhibitors. Subsequently, protein concentrations were determined using a Bicinchoninic Acid (BCA) Protein Assay Kit (Beyotime). A total of 20 or 30 μg of protein was subjected to sodium dodecyl sulfate–polyacrylamide gel electrophoresis and transferred to a 0.22 or 0.45 µm polyvinylidene difluoride (PVDF) membrane (EMD Millipore, Bedford, USA). PVDF membranes were then blocked in 5% skim milk for 2 h. Subsequently, samples were incubated with specific primary antibodies at 4 °C overnight. Following this, membranes were incubated with the appropriate secondary antibodies for 2 h at room temperature. Finally, the protein bands were visualized with enhanced chemiluminescence (ECL) Western blotting substrate (New Cell & Molecular Biotech). Information on the antibodies are provided (see Additional file 1: Table S4).

### Flow cytometry cell cycle assay

After transient transfection, U87 and U251 cells were fixed in 75% ethanol for 12 h. Subsequently, cells were stained with propidium iodide (Beyotime) for cell cycle analysis. Finally, the percentage of cells in each cell cycle phase (G0/G1, S, and G2/M) was assessed, and the results were analyzed using the ModFit LT software.

### RNA velocity and cells communication

The RNA velocity of the tumor cells was calculated using the package ‘velocity’ and ‘scVelo’ in Python. The various states of the GBM cells was mapped to show their internal transformation. The cross-talk between immunocytes and GBM cells was analyzed using the R package ‘celltalker,’ and differential ligand-receptor pairs were identified.

### Transcription factor (TF) regulatory network construction

The RcisTarget human database was downloaded from https://resources.aertslab.org/cistarget/ for transcription factor regulatory network construction. The corresponding gene ranking motif database (Hg38_refseq-r80_10kb_up_and_down_tss.mc9nr.feather, annotations_fname motifs-v9-nr.hgnc-m0.001–00.0.tbl) were downloaded from the human transcription factors list (https:/github.com/aertslab/pySCENIC/tree/master/resources), which is based on psSCENIC transcription factor regulation network. The AUCell algorithm was used to calculate the activity of each transcription factor, and the regulation module was identified according to the Connection Specificity Index (CSI). The calculation method of CSI was based on a previous article [17]. Similarly, we used the hTFtarget database to predict between TF and targets, which contains the most comprehensive data on human TF-target to date [18]. The overall activity score of the regulatory module was defined as the mean of all TF activities in the module.

### Prediction of potential drug sensitivity

The drug sensitivity information and corresponding expression level were obtained from Genomics of Drug Sensitivity in Cancer (GDSC), Cancer Cell Line Encyclopedia (CCLE), and the Cancer Therapeutics Response Portal (CTRP) (https://portals.broadinstitute.org/ctrp). The CuAS score of each cell line was calculated and grouped based on the median. The correlation between the AUC and IC50 data of multiple drugs in the cell lines was calculated by using Spearman’s correlation. The difference of the AUC value between the two groups were compared by the Wilcoxon test.

### Statistical analysis

The significance of the difference between the two groups of continuous variables was evaluated using the Wilcox rank-sum test. Spearman’s rank correlation was used to evaluate the correlation between the variables. Univariate and multivariate Cox regression and LASSO Cox regression were used to identify molecules with prognostic efficacy, and the K-M curves and log-rank tests were used to assess the survival differences between the sample groups. All computational analyses were performed by R (version 4.1.2) or Python.

## Results

### Cuproptosis characteristic gene consistent clustering to identify sample subgroups

**Fig. 1:**
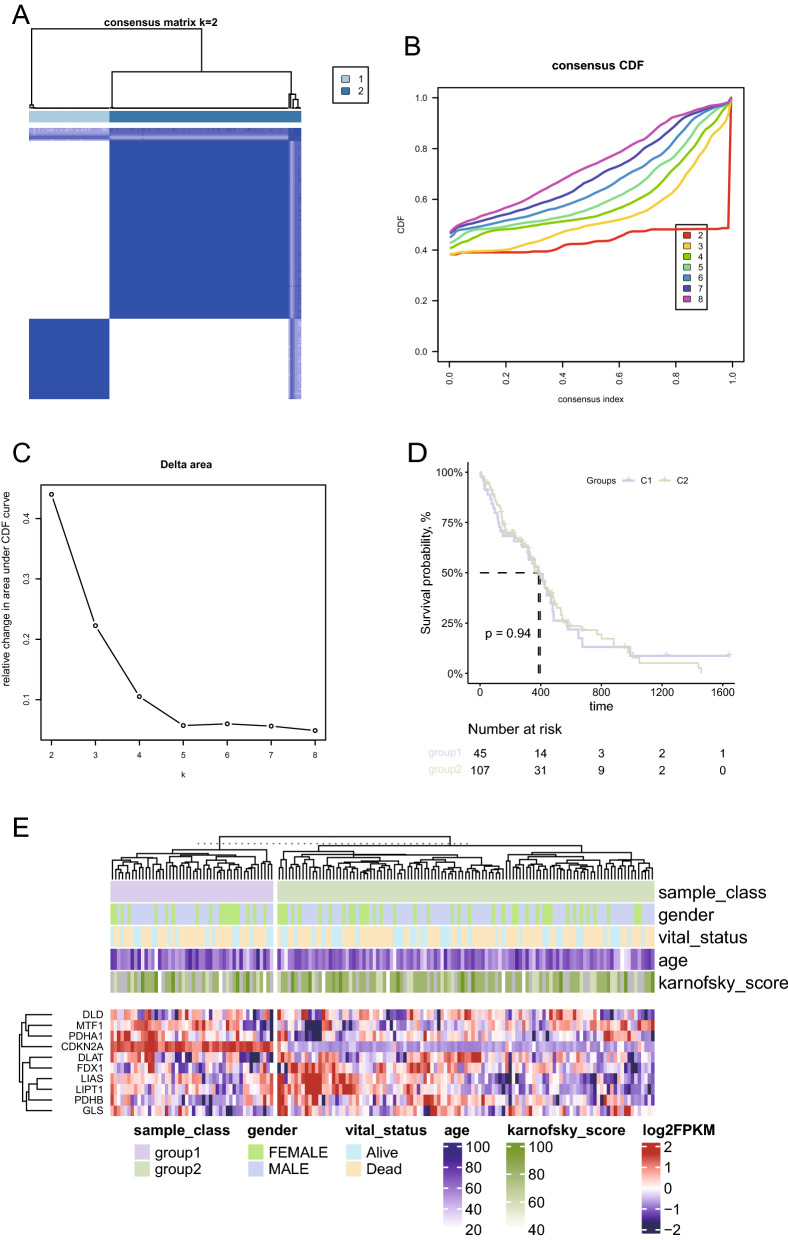
Cuproptosis characteristic gene consistent clustering to identify sample subgroups. **A** Consistent clustering matrix, where the value represents the possibility that two samples belong to the same cluster. **B** Consistent clustering accumulative distribution graph (CDF). **C** The relative change of area under the consistent clustering CDF curve. **D** Log-rank test of the overall survival difference between sample groups obtained by consistent clustering. **E** Expression pattern of cuproptosis characteristic gene in samples

**Fig. 2:**
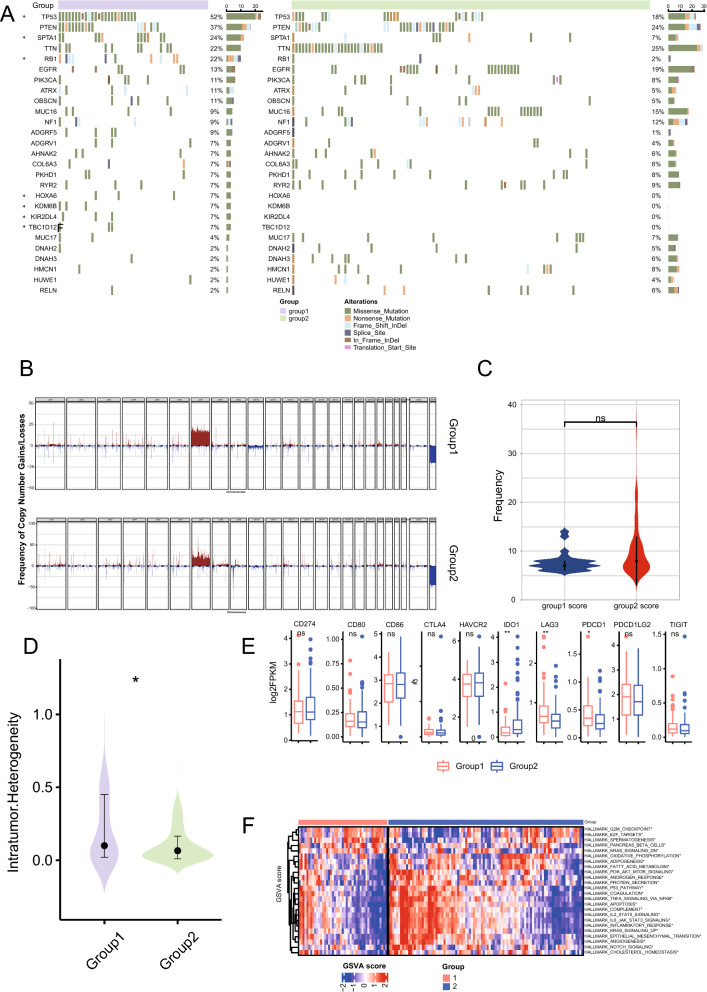
Landscape, immune checkpoint expression, and cancer hallmarks scores among the subgroups of patients with cuproptosis. **A** SNV and inDel mutation profiles in subpopulations of patients with cuproptosis. **B** Copy number variation characteristics of patients with cuproptosis. **C** Differences in the frequency of copy number variation in subsets of patients with cuproptosis. **D** Differences in intratumoral heterogeneity scores among subgroups of patients with cuproptosis. **E** Differentially expressed immune checkpoint genes in the two patient subpopulations. **F** Hallmark expression scores of cancer were significantly different between the two patient subgroups

The RNA-seq data of 169 TCGA-GBM samples were obtained, and the tumor samples were clustered into two groups based on cuproptosis genes (FDX1, LIAS, LIPT1, DLD, DLAT, PDHA1, PDHB, MTF1, GLS, and CDKN2A) through consistent cluster analysis (Fig. 1A, B, C). Significant differences were not observed in the survival between the two subgroups of samples, suggesting that these 10 genes alone may not be able to characterize the effect of cuproptosis mechanism on patient survival benefit (Fig. 1D). We also observed the expression patterns of these 10 characteristic genes in two subgroups of samples (Fig. 1E). However, significant differences were observed in the landscape of mutation, immune checkpoint expression level, and cancer hallmarks between the two subgroups. First, the SNV mutation frequencies of TP53 and other genes showed significant differences between the two types of samples (Fig. 2A). The map of CNA showed that both types of patients had significant amplification on chromosome 7 (Fig. 2B), and significant difference was not observed in total frequency of CNA (Fig. 2C). In addition, significant differences were observed in intratumoral heterogeneity between the two subgroups (Fig. 2D). Moreover, we observed a significantly different expression level of immune checkpoint genes PD-1, IDO1, and LAG3 in the two subgroups (Fig. 2E). Significant differences were observed in the cancer hallmarks of fatty acid metabolism, KRAS, P53, NOTCH, and PI3K/AKT/MTOR signaling pathway between the two subgroups (Fig. 2F).

### Construction of cuproptosis activation scoring model based on differentially expressed genes between the sample subgroups

**Fig. 3:**
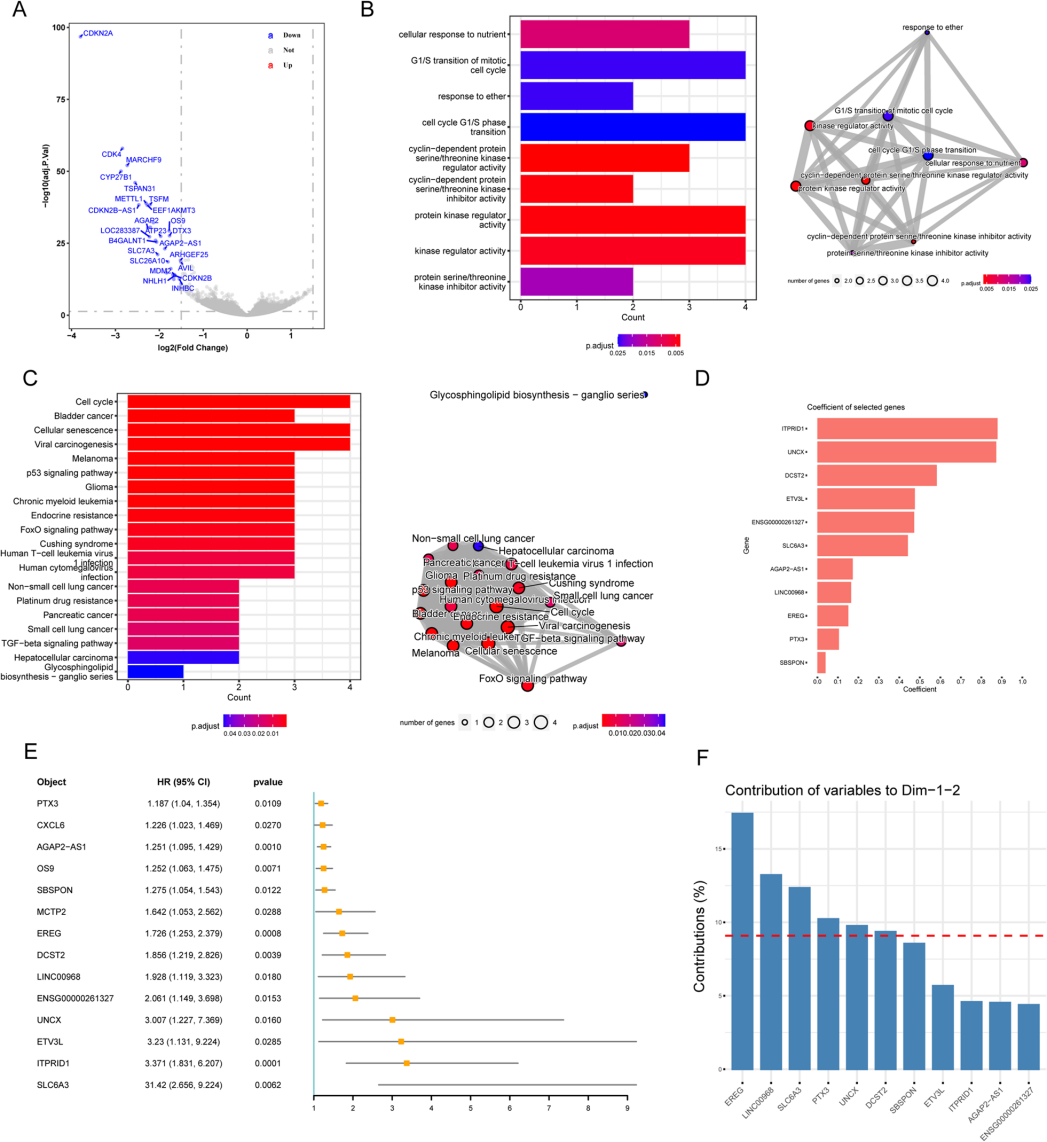
Cuproptosis Activation Scoring Model (CuAS) was constructed based on differentially expressed genes among the sample subgroups. **A** Identification of differentially expressed genes between the two groups based on DESeq2. **B** Differentially expressed genes were enriched in function by GO-BP analysis. **C** The KEGG pathway enrichment of differentially expressed genes. **D** The redundant factors were filtered by the LASSO Cox method to obtain the prognostic marker genes. **E** Univariate Cox regression was used to identify differentially expressed genes significantly associated with OS. **F** The contribution of prognostic marker genes to principal components 1 and 2 based on a principal component analysis

First, the DEGs between the two groups of samples were identified based on DESeq2 (Fig. 3A). The functions of these DEGs were enriched in the cell cycle related processes, protein kinase activity, P53 signaling pathway, and TGF-βsignaling pathway (Fig. 3B, C). In the TCGA-GBM sample set, we identified 14 candidate prognostic marker genes that were significantly associated with the OS of patients based on univariate Cox regression (Fig. 3E) and further filtered the redundant factors using LASSO Cox to obtain 11 prognostic marker genes (Fig. 3D). Based on the PCA of these 11 genes, their contribution to principal components 1 and 2 were used as coefficients (Fig. 3F) to construct CuAS.

### The prognostic efficacy of CuAS

**Fig. 4:**
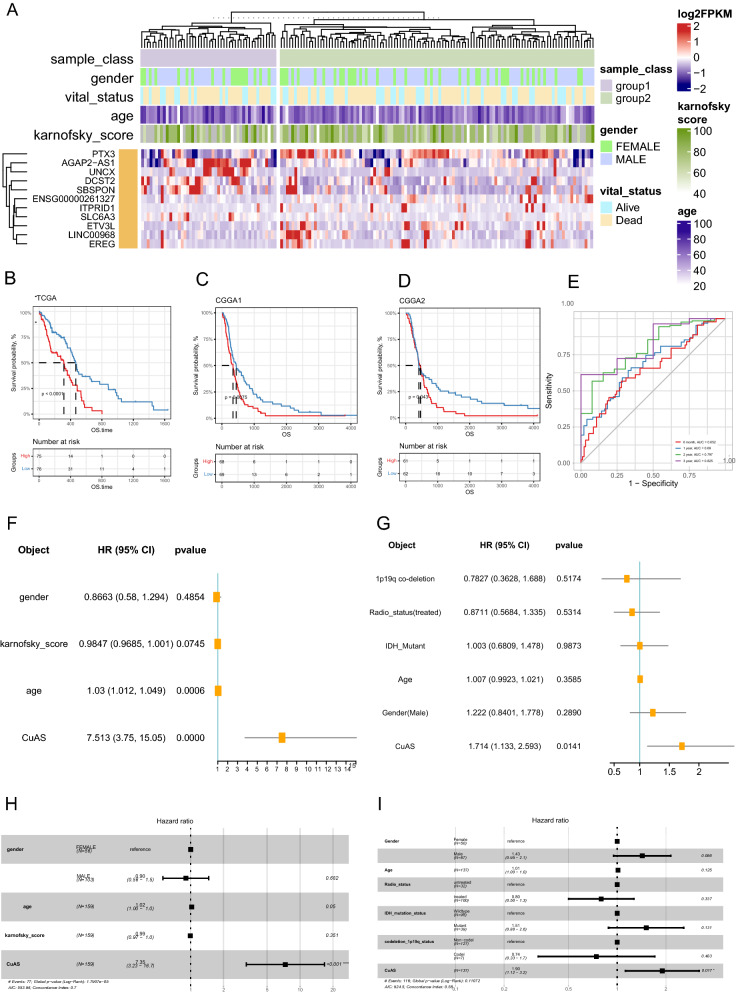
Prognostic efficacy verification of CuAS. **A** Expression patterns of 11 prognostic marker genes in TCGA samples. **B** Differences in overall survival among CuAS score groups in TCGA samples. **C** Significant differences in overall survival among CuAS score groups in validation set CGGA1. **D** Significant differences in overall survival among CuAS score groups in validation set CGGA2. **E** The predictive efficacy of CuAS scores in TCGA samples for patient survival. Univariate Cox regression was used to evaluate the prognostic efficacy of CuAS scores and clinical features in (**F**) TCGA samples and (**G**) validation set CGGA2. Multivariate Cox regression was used to evaluate the independence of prognostic efficacy of CuAS scores and clinical features in (**H**) TCGA samples and (**I**) validation set CGGA2

In the training set, we scored the samples based on these 11 genes and the results of the PCA (Fig. 4A). We found that patients with higher CuAS scores had significantly worse OS (Log-rank P < 0.0001, Fig. 4B) and 6 month AUC (95%CI) = 0.625 (0.608–0.643), 1 year AUC (95%CI) = 0.69 (0.646–0.732), 2 year AUC (95%CI) = 0.797 (0.735–0.852), 3 year AUC (95%CI) = 0.825 (0.765–0.885) (Fig. 4E). In addition, two validation sets from the CGGA database showed that CuAS had stable prognostic efficacy (Log-rank P = 0.0075, Fig. 4C, Log-rank P = 0.0043, Fig. 4D). Univariate Cox regression analysis was performed on CuAS and multiple clinical features to evaluate the independence of the prognostic efficacy of CuAS. The results showed that CuAS was significantly associated with patient prognosis (HR (95% CI) = 7.51 (3.75, 15.05)) (Fig. 4F). Consistent results were also observed in the independent validation sets (HR (95% CI) = 1.74 (1.133, 2.593)) (Fig. 4G). Furthermore, we constructed a multivariate Cox regression model for CuAS and multiple clinical features and found that CuAS could still serve as an independent prognostic factor (HR (95% CI) = 7.35 (3.23, 16.7)) (Fig. 4H), HR (95% CI) = 1.90 (1.12, 3.2)) (Fig. 4F)).

### Epiregulin (EREG) was an oncogenic gene that can influence immunity and cuproptosis

**Fig. 5:**
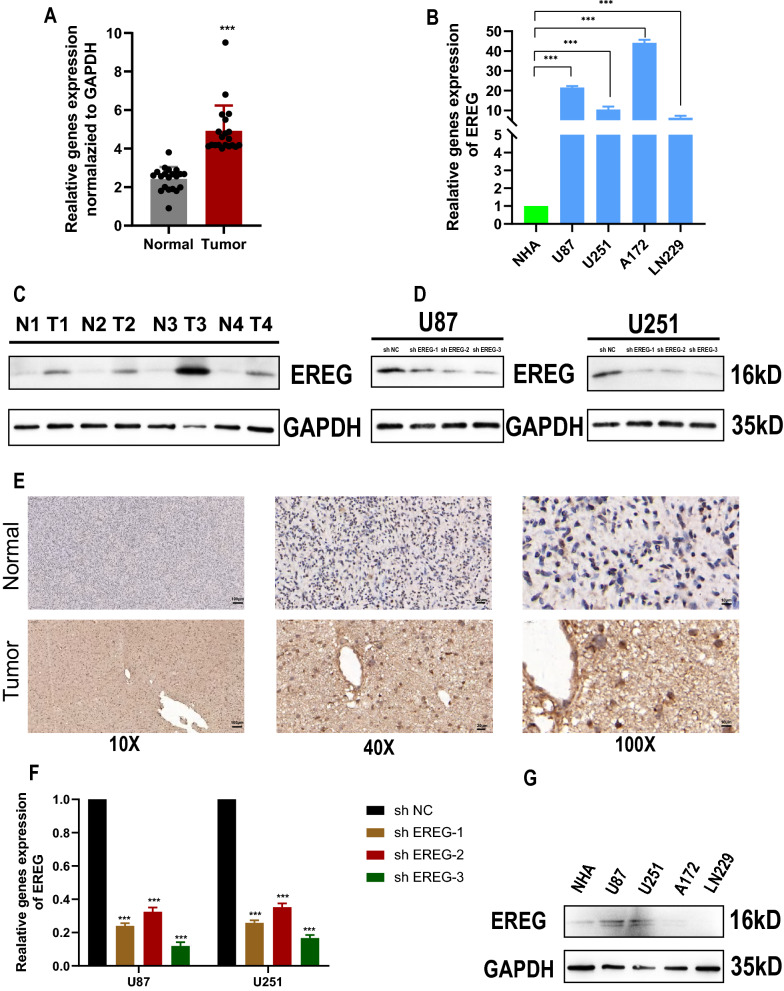
mRNA and protein expression of EREG. **A** mRNA expression of normal and tumor tissues. **B** mRNA expression of NHA and GBM cell lines. **C** Protein expression of normal and tumor tissues. **D** Western blot was used to detect knockdown inefficiency of EREG in the U87 and U251 cell lines. **E** The protein expression of EREG in normal and tumor tissues was detected by immunohistochemistry. **F** qRT-PCR was used to detect knockdown inefficiency of EREG in the U87 and U251 cell lines. **G** Protein expression of NHA and GBM cell lines.*P < 0.05, **P < 0.01, ***P < 0.001. Error bars indicate the mean ± SD

**Fig. 6:**
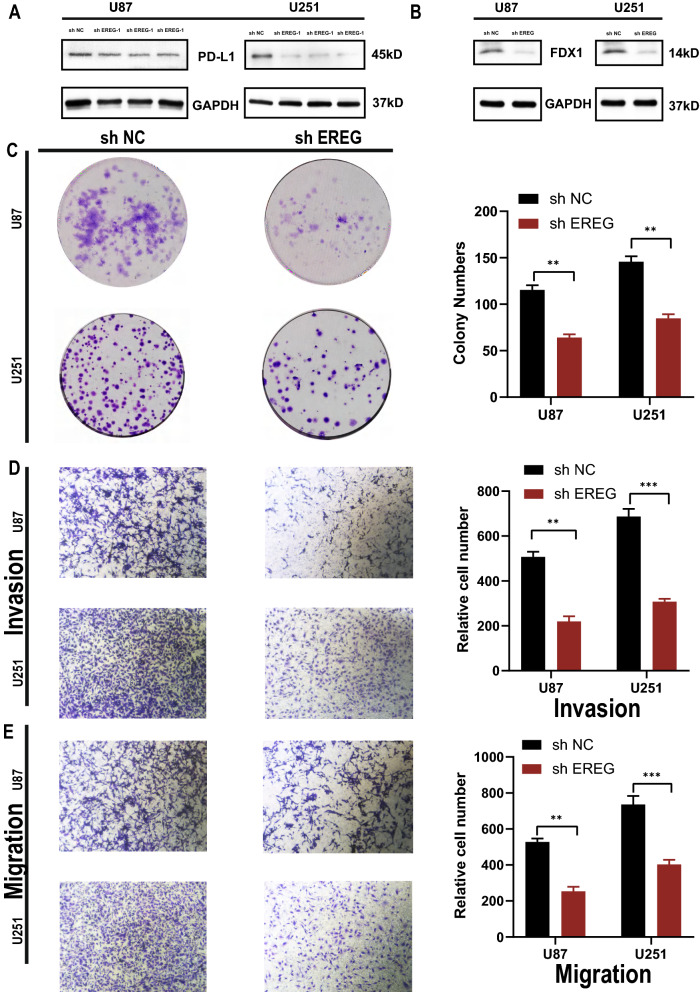
EREG function analysis. **A** PDL1 expression of EREG-KD and EREG-NC in the U87 and U251 cell lines. **B** FDX1 expression of EREG-KD and EREG-NC in the U87 and U251 cell lines. **C** Colony formation assays were performed on U87 and U251 cell lines. **D** Invasion ability assays were performed on U87 and U251 cell lines by transwell. **E** Migration ability assays were performed on U87 and U251 cell lines by transwell.*P < 0.05, **P < 0.01, ***P < 0.001. Error bars indicate the mean ± SD

**Fig. 7:**
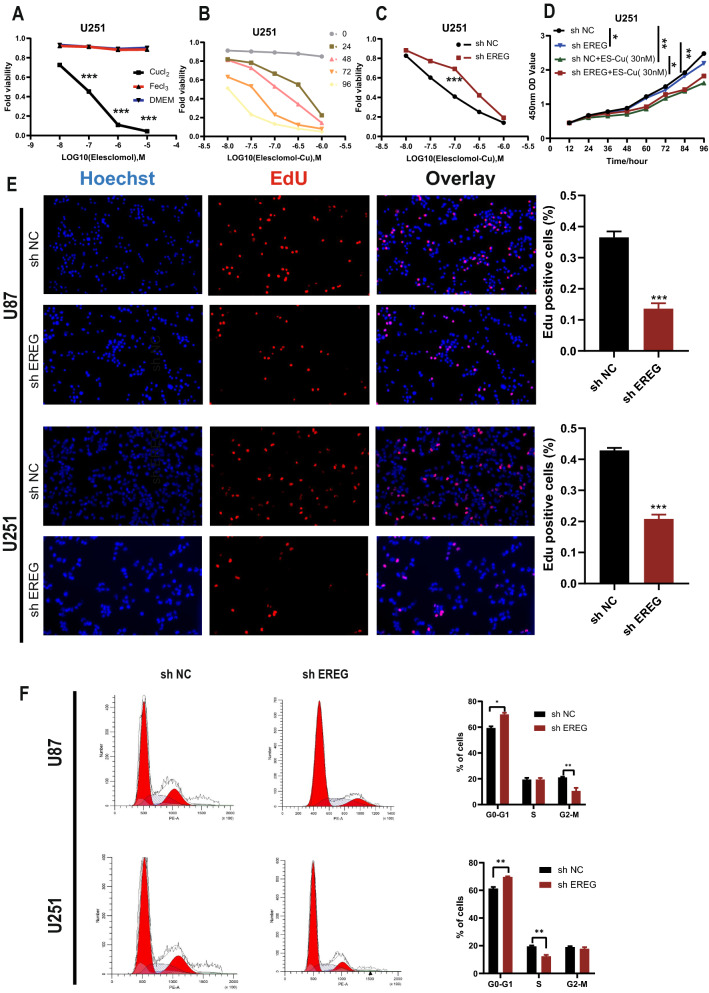
EREG proliferation analysis. **A** Viability of cells (U251) after treatment with elesclomol with or without 10 mM of indicated metals. **B** V iability of U251 cells was assessed at the indicated times after elesclomol-Cu (1:1 ratio) treatment. ES, elesclomol. (**C**) Viability of shNC and shEREG in U251 cells was assessed at the indicated times after elesclomol-Cu (1:1 ratio) treatment. ES, elesclomol. **D** Cell viability of U251 cells after knocking down EREG was determined using CCK8 assays with or without the presence of 20 nM elesclomol-Cu. **E** U87 and U251 cells were treated with EdU for 6 h prior to click reaction. Data analysis was performed to calculate the signal intensity in EdU-positive cells based on individual DAPI signal and is displayed in the right graph. **F** Cell cycle distribution was analyzed by PI staining in U251 and U87 cells of shNC and shEREG. *P < 0.05, **P < 0.01, ***P < 0.001. Error bars indicate the mean ± SD

The EREG mRNA expression levels were high in tissues and multiple glioma cell lines (Fig. 5 A, B). We used Western blot (Fig. 5C) and IHC staining (Fig. 5E) to detect the EREG protein expression levels in tumors and normal tissues, and we found that the protein expression levels of EREG in tumors were higher than that in normal tissues. Additionally, we found that the protein expression levels of EREG in glioma cell lines were higher than that in normal astrocytic cell lines (Fig. 5G). Subsequently, we constructed knockdown stable cell lines of EREG and verified the knockdown effects on mRNA (Fig. 5F) and protein levels (Fig. 5D). Functional experiments showed that EREG knockdown (KD) can significantly inhibit the proliferation detected by Edu exepriments (Fig. 7E), invasion (Fig. 6D), migration (Fig. 6E), and colony forming ability (Fig. 6C) of tumor cells. Additionally, flow cytometry cell cycle assays suggested that EREG KD significantly inhibited cell cycle progression from the G0/G1 phase to the S phase (Fig. 7F). To explore the relationship between EREG and immune infiltration, we detected the expression level of PDL1 in EREG-KD group and found that PDL1 also decreased (Fig. 6A). To explore the relationship between EREG and cuproptosis, we performed the different treatment gradients of Cu-Elesclomol(ES) (1:1) on U251 cell lines and found that cell viability decreased with increasing time, and the effect of ES-Cu required a specific concentration range (5–50 nM) (Fig. 7A, B). Subsequently, we treated tumor cells with the same concentration (30 nM) with ES-Cu, and observed cell viability at 0, 12, 24, 36, 48, 60, 72, 84, and 96 h (Fig. 7D). It was found that the proliferation rate of the treated cells decreased significantly compared with the cells that were not treated with ES-Cu. Then, the same treatment was performed on the shEREG and shNC groups and found that the proliferation rate of the two treated groups decreased significantly when compared with the shEREG and shNC groups that were not treated with ES-Cu; however, the reduction rate of the proliferation of the shEREG group was higher than that of the shEREG group with ES-Cu treatment, indicating that EREG can influence cell proliferation by affecting the process of cuproptosis (Fig. 7C, D). Therefore, we detected the protein expression level of FDX1 in the shEREG and shNC groups. The results showed that FDX1, the core regulatory protein in cuproptosis, was down-regulated in the shEREG group (Fig. 6B). Based on the above results, we believe that EREG is an oncogenic gene that can affect immunity by influencing the expression level of PDL1 and is closely related to the process of cuproptosis.

### Single cell transcriptome analysis of CuAS patterns

**Fig. 8:**
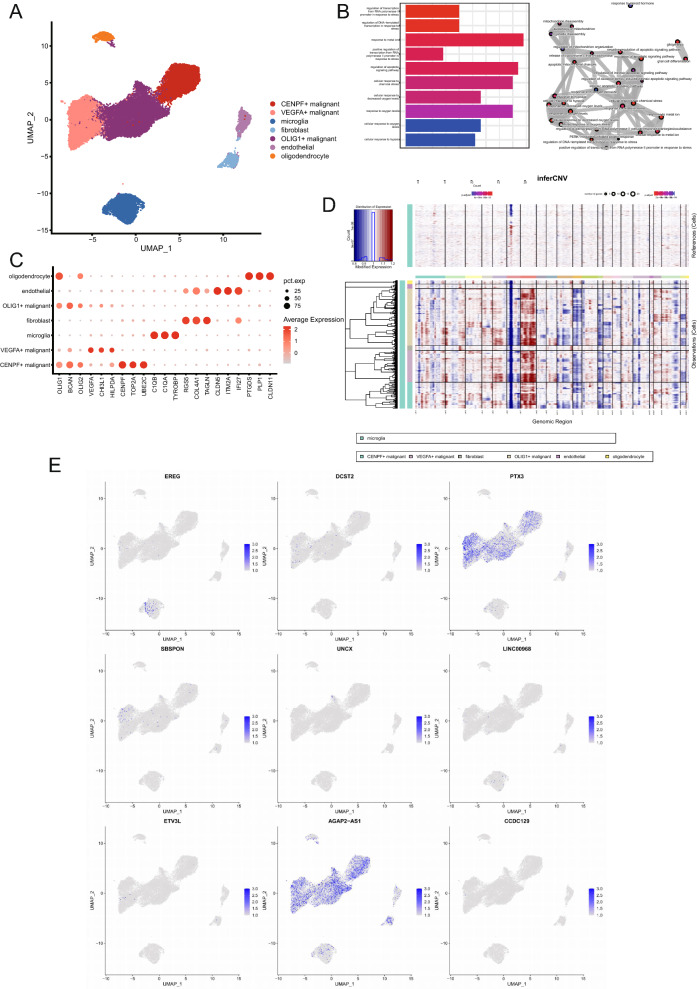
Single cell transcriptome analysis of CuAS patterns. **A** Clustering and annotation of single cell data. **B** Functional enrichment analysis based on hypergeometric tests and functional enrichment network constructed based on term similarity. **C** Expression distribution of marker genes of each cell type in each subpopulation. **D** Based on the copy number spectrum obtained by inferCNV, the origin of cell clone evolution was determined. **E** The expression distribution of characteristic genes

Based on the downloaded single cell data (GSE173278, 29339 cells,10X Genomics platform), R Package Seurat was used to process the data. The expression profile was transformed by Log10, and 2,000 highly mutated genes were identified based on the VST method. Subsequently, principal component analysis and dimensionality reduction visualization were performed using UMAP. As a strong batch effect was observed, Harmony was used for batch correction (see Additional file 2: Fig. S1). Follow-up analysis was conducted based on the corrected data. The default parameters were used for clustering, and the meaning reference of analysis results based on known markers (SingleR was BP and HPCA) for cell type annotation (Fig. 8A). The cells in the GBM samples were divided into 7 categories, three malignant cell (OLIG1 + malignant, n = 11637; VEGFA + malignant, n = 6446; CENPF + malignant, n = 5363), microglia (n = 3219), fibroblasts (n = 1020), endothelial cells (n = 919), and oligodendrocytes (n = 735) (Fig. 8C). CuAS was calculated by using the previous model coefficients, but several cuproptosis characteristic genes were not detected in single cell data, and the expression level of many characteristic genes was undetectable (see Fig. 8E and Additional file 2: Fig. S2). A small number of cells (approximately 2800 cells) with a high CuAS score accounted for less than 10% of the whole cells and were distributed in multiple cell subpopulations. Most of the subpopulations contained less than 5% of cells with high CuAS, and 1132 cells with high CuAS were present in VEGFA + malignant cells (hypergeometric test, p value < 0.05). Trajectory inference of tumor cells are depicted (see S Additional file 2: Fig. S3). Moreover, the ancestor clone was determined based on CNV in combination with the idea of clone evolution, so as to determine the evolutionary relationship between cells more accurately. Based on CNV, we found that the OLIG1 + malignant cell may be the ancestor clone (Fig. 8D). Furthermore, in order to better explain the functional role of CuAS at the single cell level, the functions of VEGFA + malignant cells were observed and functional enrichment analysis was conducted based on specific up-regulated genes obtained by differential expression analysis. Pathways are mainly enriched in pathways related to hypoxia and oxidative stress (Fig. 8B).

### Differential activation of transcription factors between high and low CuAS

**Fig. 9:**
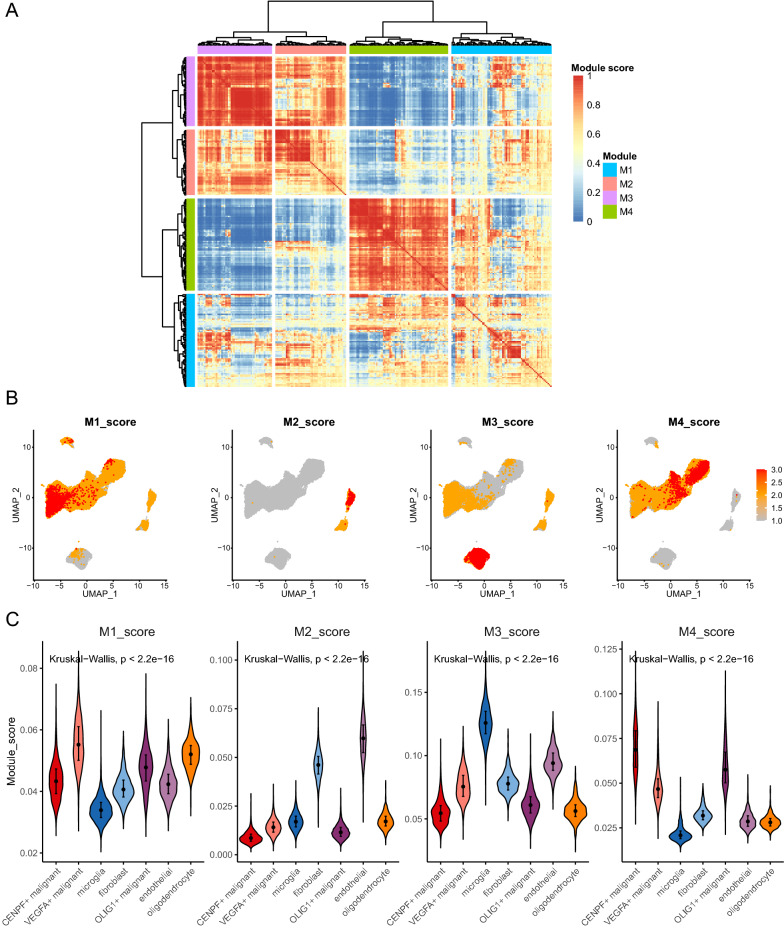
Transcription factors activity analysis with single cell data. **A** TF module was identified by the connection specificity index (CSI). **B** Distribution of TF module scores in cell subsets. **C** Significant differences of TF module scores in cell subsets

Annotated files of human transcription factors were obtained from the RcisTarget database and the list of human transcription factors were downloaded. Transcription factor regulatory network was constructed using pySCENIC. Subsequently, the AUCell algorithm was used to calculate the activity of each transcription factor, and according to the CSI between the different transcription factors, four regulatory modules were identified (Fig. 9A). Module score was performed for each cell sample. We explored the association between cell type and module score, which revealed that the score of Module1 was significantly higher in VEGFA + malignant cells, while the score of Module2 was significantly higher in the endothelial cell subset. The score of Module3 was significantly higher in the microglia cell subset, while the score of Module4 was higher in the CENPF + malignant cells and partial OLIG1 + malignant cells, which reflected the differences of TF activated by different malignant cell subsets (Fig. 9B, C). Similarly, the results of the hTFtarget database showed that transcription factors such as FOSL2, JUND, NFIC and PBX3 were highly active in the VEGFA + malignant subgroup (see Additional file 2: Fig. S4). In particular, we previously observed that cells with high CuAS scores were concentrated in VEGFA + malignant subsets, showing the potential association between CuAS scores and Module1.

### Correlation between CuAS and immune microenvironment

**Fig. 10:**
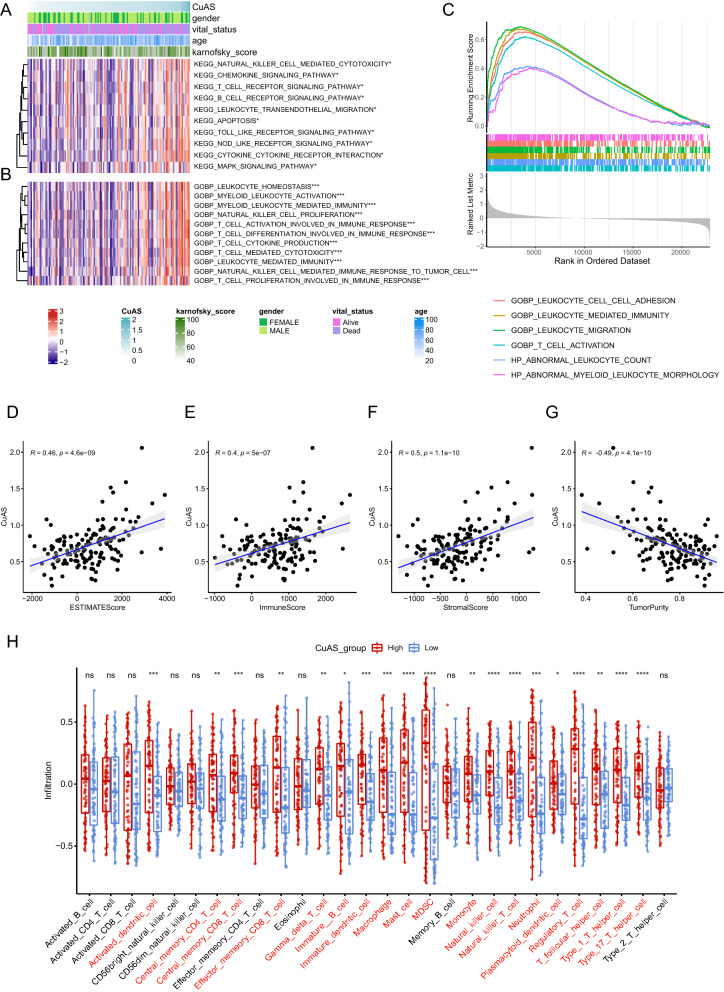
Correlation between CuAS and immune microenvironment composition. KEGG pathway (**A**) and Go-BP term (**B**) enriched differentially between high CuAS groups and low CuAS groups in TCGA-GBM samples. **C** Differential enrichment signature between high and low CuAS groups based on GSEA enrichment identification. **D**–**G** Correlation of CuAS with ESTIMATE score (**D**), immunity score (**E**), stromal score (**F**), and tumor purity (**G**) inferred by ESTIMATE algorithm. **H** Association of CuAS with immune cell infiltration

First, GO/KEGG enrichment analysis based on GSVA algorithm was performed on TCGA-GBM tumor samples, and immune-related pathways were differently enriched between the high and low CuAS groups, including T cells, NK Cell, B cell signal, chemokine signal, cytokine interaction, and other pathways (Fig. 10A). GO enrichment also showed that the activation differentiation and proliferation of T cells, NK cell proliferation, cytotoxic reaction, and other characteristics were highly enriched when CuAS scores were high (Fig. 10B). In addition, we identified differentially enriched signatures between the high and low CuAS groups based on GSEA enrichment analysis and also captured immune reaction processes such as leukocyte adhesion migration and T cell activation (Fig. 10C), indicating that the mechanism of cuproptosis is closely related to immune reaction process. Further, we calculated the immune and stromal components by using ESTIMATE, and it was observed that CuAS was significantly positively correlated with the stromal, immune, and ESTIMATE scores, while it was significantly negatively correlated with tumor purity (Fig. 10D–G), which suggested the association between cuproptosis and immunity. By using GSVA to calculate immune cell infiltration, we found that cuproptosis was significantly correlated with various types of immune cell infiltration, including activated DC and NK cells. (Fig. 12H). In addition, CIBERSORT and xCell methods were used to calculate various immune cell infiltrates, which revealed similar results (see Additional file 2: Fig. S5).

### Specific cell communication was different between high and low CuAS groups

**Fig. 11:**
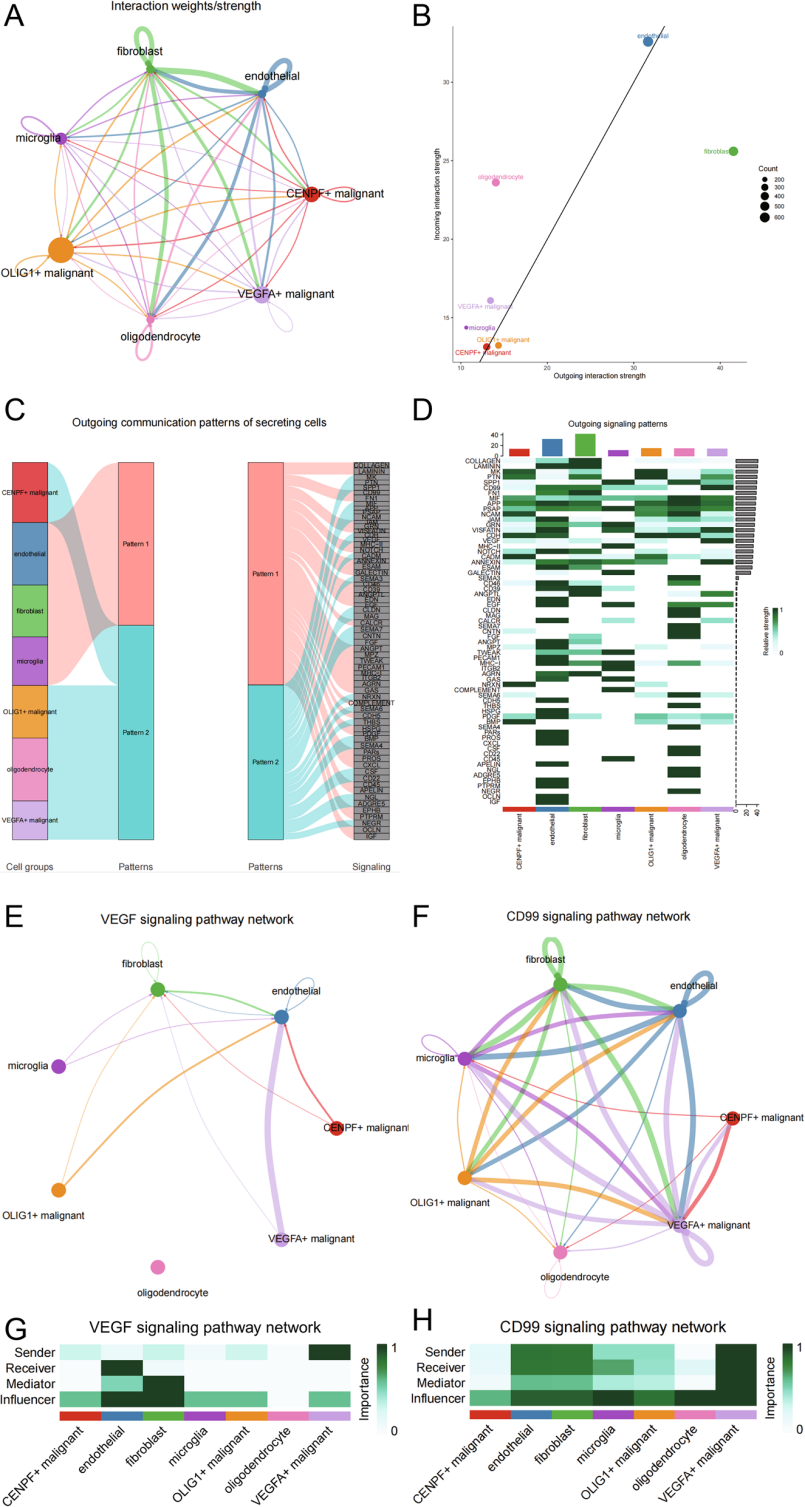
Cell communication analysis. **A** A global cellular communication network; **B** Each cell subpopulation mainly played the role of incoming or outgoing; **C** Intercellular communication based on NMF method can be divided into two modes; **D** Main signaling pathways (**E**, **F**) associated with VEGFA + malignant cell subpopulation and their cell communication networks; **G**, **H** Primary originators and influencers of key signaling pathways

**Fig. 12:**
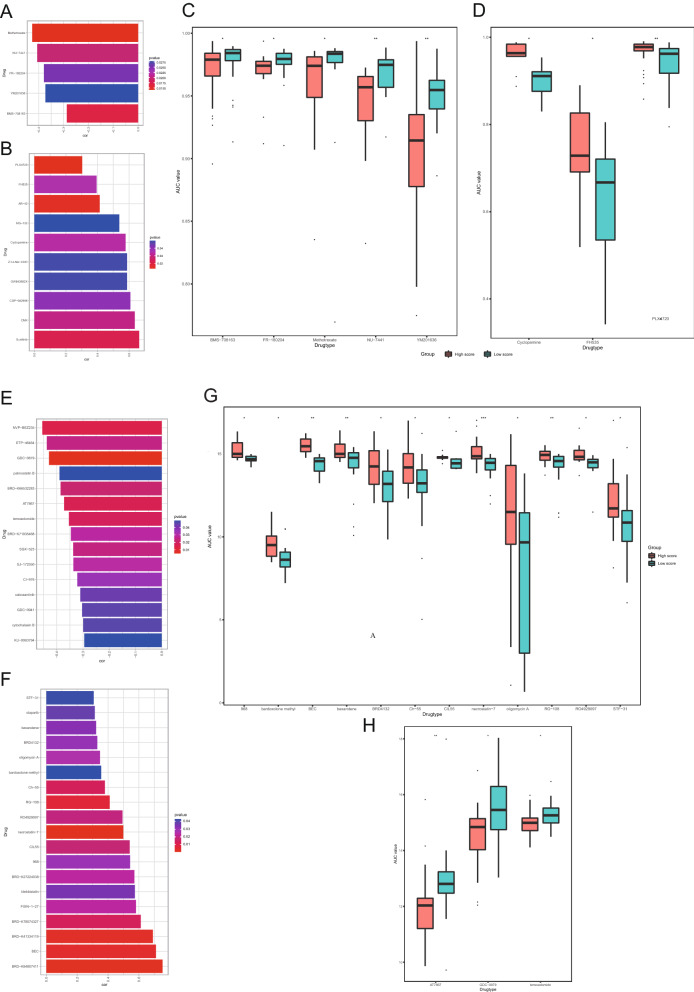
Drug sensitivity between different signature groups of GDSC and CTRP. **A** Negative correlation between signature score in GDSC and drug AUC (P < 0.05); **B** Positive correlation between signature score in GDSC and drug AUC (P < 0.05); **C** Differences in signature scores of GDSC cell lines with significant negative correlation under different drug treatments; **D** Differences in signature scores of cell lines with significant positive correlation under different drug treatments in GDSC. **E** Negative correlation between signature score and DRUG AUC in CTRP (P < 0.05); **F** Positive correlation between signature score in CTRP and drug AUC (P < 0.05); **G** Difference in signature scores of all cell lines in CTRP with significant negative correlation under different drug treatments; **H** Differences in signature scores of all cell lines in CTRP with significant positive correlation under different drug treatments

**Fig. 13:**
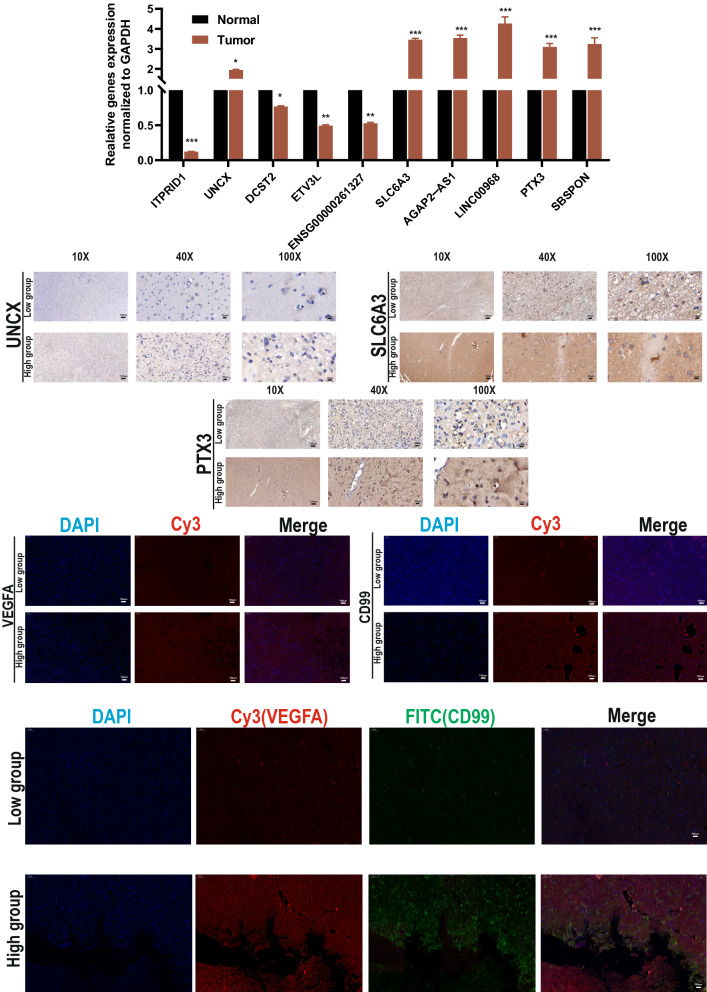
Experimental validation of model genes. **A** The genes of model expression were detected by qRT-PCR. Immunohistochemistry was performed on UNCX (**B**), SLC6A3 (**C**), and PTX3 (**D**) in high or low CuAS group. Immunofluorescence detected the expression of VEGFA (**E**) and CD99 (F) in tissue samples with high and low CuAS group. **G** Coexpression of VEGFA and CD99 in tissue samples with high and low CuAS group. *P < 0.05, **P < 0.01, ***P < 0.001. Error bars indicate the mean ± SD

As the high CuAS cells were significantly enriched in VEGFA + malignant cells, we mainly analyzed the difference in communication and function between the VEGFA + malignant and other cells. Extensive cell communication was observed in each cell subpopulation (Fig. 11A). Furthermore, by distinguishing between the incoming and outgoing signals, we found that fibroblasts are the dominant signaler of outgoing signaling, and VEGFA + malignant cells are the signal receivers (Fig. 11B). Furthermore, we identified two patterns of cell subpopulations in outgoing signaling, in which VEGFA + malignant cells belonged to Pattern 2 and corresponding pathways included VEGF, FGF, CDAM, CD22, ADGRE5, and other malignant progression related pathways (Fig. 11C). Meanwhile, we analyzed the dominant signaling pathway of each cell and found that VEGFA + malignant cells are not only involved in the signaling pathway of VEGF, but also in the CD99 signaling pathway, which was not proposed in the non-negative Matrix Factorization (NMF) analysis (Fig. 11D). The CD99 signaling pathway plays an important role in tumor progression and transendothelial migration of cancer cells. VEGF and CD99 signaling pathways were further analyzed, and it was found that VEGFA + malignant cells are the dominant signalers of VEGF signals, and the cell subsets that were affected are mainly the endothelial cells and fibroblasts, both of which are important components of angiogenesis (Fig. 11 E, G). In addition, VEGFA + malignant cells are the dominant signaler, receiver, and influencer of CD99 signaling pathways, indicating that CD99 signaling pathways can occur as feedback loops (Fig. 11 F, H). Meanwhile, endothelial and fibroblast cells are also affected by CD99 signaling pathways, suggesting that VEGEA + malignant cells can influence transvascular endothelial migration (Fig. 12). The immunofluorescence detection of tissue samples with high and low CuAS showed that VEGFA and CD99 were also highly expressed in tissues with high CuAS. The results were opposite in tissues with low CuAS (Fig. 13E, F, G), which provided a new idea for the intervention of cuproptosis-related tumor cells.

### CuAS is associated with prognosis of immunotherapy

Based on all the immunotherapy data searched, we observed the ability of the CuAS score in predicting the prognosis and efficacy in the immunotherapy cohort. Phs001493 (Renal cell carcinoma, Anti-PD1 therapy) and PRJEB23709_ipiPD1 (Melanoma,anti-CTLA4 & AMP; Anti-pd1 dual antibody therapy) were significantly associated with worse prognosis (see Additional file 2: Fig. S6B and D). For a patient’s Progression Free Survival (PFS), we found that NCT02684006 (kidney cancer, anti-PDL1 treatment) was significantly associated with worse prognosis (see Additional file 2: Fig. S6E). Therefore, high CuAS patients may benefit from immunotherapy.

### Potential targeted drugs for high CuAS glioblastoma cells

The expression data of cell lines were extracted from three databases: GDSC, CCLE, and CTRP. A lower AUC value represents a higher sensitivity to drugs. Using the AUC data provided by these databases, multiple drugs with a negative correlation between the AUC and signature were found in GDSC, such as methotrexate, BMS -708163, YM201636, FR -180204, and NU − 7441 (Fig. 12A). A variety of drugs with positive correlations were also found, such as cyclopamine (Fig. 12B). These drugs showed significant differences in the AUC between the groups of high and low signature (Fig. 12 C, D). No drugs with an IC50 significantly correlated with signature were found in CCLE, while drugs with a significant AUC (KU -0063794, cytochalasin B, GDC − 0941, cabozantinib, CI − 976, SJ -172550, SGX − 523, BRD − K71935468, temozolomide, AT7867, BRD-K66532283, palmostatin B, GDC-0879, ETP-46464, and NVP-BEZ235) negatively correlated with signature were found in CTRP (Fig. 12E, F). These drugs were found in the AUC values of the high and low signature groups were significantly different in CTRP (Fig. 12 J, H). Therefore, high CuAS samples are likely to be sensitive to these compounds, and these compounds may be novel treatment options for GBM.

### Experimental validation of model genes

The genes expression levels in the model were detected by qRT-PCR, and the results showed that they were highly expressed in 20 pairs of tumor and normal tissues (UNCX, SLC6A3, AGAP2-AS1, LINC00968, PTX3 and SBSPON), while ITPRID1, DCST2, ETV3L, and ENSG00000261327 were down-regulated (Fig. 13A). According to the corresponding PCR results, we divided the tissue samples into high and low CuAS groups. IHC staining was performed on UNCX, SLC6A3, and PTX3, and it was found that the protein expression of the high CuAS group was higher than that of the low CuAS group (Fig. 13B, C, D).

## Discussion

Copper is an essential cofactor in all organisms; however, it is toxic for cells when concentrations of copper exceed thresholds maintained by an evolutionarily conserved homeostasis mechanism [19, 20]. In fact, it is not known how excessive copper can induce cell death. However, the Broad Institute has currently identified a new mechanism that is different from known cell death: cuproptosis [8]. Cuproptosis is a kind of cell death that is dependent on mitochondrial respiration. Copper directly binds to lipoylated components of the tricarboxylic acid cycle. Afterwards, aggregation of these copper-bound, lipoylated mitochondrial proteins and subsequent Fe-S cluster protein loss trigger proteotoxic stress and a distinct form of cell death [19–22]. Cuproptosis is involved in cell death, and the Broad Institute paper suggests that drugs that inhibit mitochondrial respiration may be a strategy against disease [19–22]. In addition, many mitochondrial proteins have a high degree of respiration function in various cancer cells [23]. Thus, copper ion metal carriers may be a new method for cancer treatment.

To the best of our knowledge, this study was the first paper to comprehensively analyze the association between copper-induced cell death and GBM by combining scRNA-seq and bulk RNA-seq data. First, we identified two sample subgroups based on the characteristic genes of cuproptosis. We found that immune checkpoint genes (PD-1, IDO1 and LAG3) and cancer hallmarks (fatty acid metabolism, KRAS, P53, NOTCH, and PI3K/AKT/MTOR signaling pathway) showed significant differences between the two subgroups. Immune checkpoint is a kind of immunosuppressive molecule, which can regulate the intensity and breadth of the immune response, to avoid the damage and destruction of normal tissues. In the process of tumor occurrence and development, immune checkpoint has become one of the main reasons for immune tolerance. Subsequently, we constructed CuAS based on the differential genes of subgroups, which contained 11 genes, including 8 coding genes and 3 non-coding genes. EREG was the gene with the largest contribution coefficient to the principal component, so we focused on EREG. EREG is a 19-kDa peptide hormone that belongs to the Epidermal Growth Factor (EGF) family of peptide hormones [24]. Epiregulin binds to the EGF receptor (EGFR/ErbB1) and ErbB4 (HER4) and stimulates signaling of ErbB2 (HER2/Neu) and ErbB3 (HER3) through ligand-induced heterodimerization with a cognate receptor [24]. EREG possesses a range of functions in both normal physiologic states as well as in pathologic conditions. EREG contributes to inflammation, wound healing, tissue repair, and oocyte maturation by regulating angiogenesis and vascular remodeling and by stimulating cell proliferation [24]. Deregulated EREG activity appears to contribute to the progression of a number of different malignancies, including cancers of the bladder, stomach, colon, breast, lung, head and neck, and liver [2, 7, 24]. EREG is also associated with imaging omics as an important prognostic gene and MRI parameters revealed that hemodynamic abnormalities were associated with the expression level of the mTOR‐EGFR pathway in patients with GBM [25]. Rab27b promotes the proliferation of adjacent cells and radio-resistance of highly malignant GBM cells through EREG-mediated paracrine signaling after irradiation [26]. Furthermore, EREG activates the extracellular signaling-related kinase/MAPK pathway in GBM, suggesting that the inhibition of the EREG-EGFR interaction may be a strategy for EREG-overexpressing patients with GBM [2]. In our study, we detected EREG mRNA expression and protein levels in tissues and multiple glioma cell lines. IHC staining revealed that the EREG protein expression in tumors was higher than that in normal tissues; the result of WB also showed similar results. Knockdown of EREG can inhibit the proliferation, invasion, and migration of tumor cells. EGFR and PDL1 expression of protein were down-regulated after knockdowning of EREG. Moreover, we explored if EREG could influence the process of cuproptosis. Cell vitality assay demonstrated that only the coexistence of Cucl_2_ and ES can influence the cell vitality and that other metals had no effect. The effect of ES-Cu required a specific concentration range (5 nM-50 nM). shEREG can revert the cell vitality that is influenced by cuproptosis. Therefore, we detected the protein expression of FDX1 in the shEREG and shNC groups. The results showed that FDX1, the core regulatory protein in cuproptosis, was down-regulated in the shEREG group.

Combined with the single cell transcriptome, the model of cuproptosis was analyzed, and the GBM sample cells were divided into seven types, including three types of malignant cells (OLIG1 + malignant, VEGFA + malignant, and CENPF + malignant). OLIG1 and other oligodendrocyte markers were highly expressed in OLIG1 + malignant cells, which may be oligodendrocyte progenitor glioma mother cells. VEGFA, CHI3L1, and other angiogenesis related markers were highly expressed in VEGFA + malignant cells, which may have a strong ability to induce local angiogenesis and may be associated with invasion/metastasis. CENPF + , TOP2A, UBE2C, and other markers are associated with the cell cycle and may be mesenchymal glioma blasts, which may be associated with tumor proliferation/invasion [27, 28]. Others types observed were microglia, fibroblasts, endothelial cells, and oligodendrocytes. High CuAS was found in VEGFA + malignant cells. Based on CNV [29], OLIG1 + malignant cells were the ancestor clones. The function of VEGFA + malignant cells demonstrated that the pathways were mainly enriched in those related to hypoxia and stress, which is also consistent with the fact that cuproptosis is mitochondrion-dependent programmed cell death. Activated cells with high CuAS scores based on differences between high and low CuAS transcription factors were concentrated in the VEGFA + malignant cell subpopulation, reflecting the potential association between CuAS scores and Module1. The VEGF and CD99 signaling pathways were significantly enriched in high CuAS cells. VEGF specifically binds to Fltl and KDR/Flkl on the surface of endothelial cells, resulting in a variety of biological effects [30]. VEGF is closely associated with angiogenesis and development [31]. VEGF plays an important role in all stages of tumor formation, inducing the production of a large number of proteolytic enzymes, reducing the basement membrane of the host blood vessels, weakening the barrier effect, increasing the permeability of blood vessels, promoting a large amount of fibrinogen exudation, and forming a new matrix necessary for tumor adhesion and migration [30, 31]. Angiogenesis is determined by the growth and metastasis of solid tumors. VEGF degrades extracellular matrix by inducing endothelial cells to express protease, resulting in metastasis, proliferation, and angiogenesis [32]. CD99 is abnormally expressed in many different types of tumors, and plays an important role in the diagnosis, development, metastasis, and prognosis, mainly affecting the invasion and metastasis of tumor cells [33]. Immunofluorescent detection of tissue samples with high and low CuAS showed that VEGFA and CD99 were also highly expressed in tissues with high CuAS, and the results were opposite in tissues with low CuAS, which provided a new idea for us to intervene in cuproptosis-related tumor cells.

Immunotherapy is essential in tumor treatment. Despite the lack of specific immune cohort verification for glioma, several other tumor immune cohorts have shown the possibility of treatment for patients with high CuAS. Considering that EREG may affect the expression of PDL1 and the immune process, we believe that immunotherapy may have therapeutic opportunities for patients with high CuAS. Chemotherapy is also the first line of treatment for glioma. We predicted the potential targeted drugs for high CuAS GBM cells. Methotrexate can be used to treat GBM owing to several factors such as the upregulation of CD73 [34]. Pharmacological inhibition of DNA-PKcs with the DNA-PKcs inhibitor NU7441 reduced GSC tumorsphere formation [35] mTORC1/2 inhibitors of KU - 0063794 can inhibit PI3K-Akt-mTOR signaling in glioblastoma and reduce cell proliferation [36]. The PI3K inhibitor GDC-0941 enhances radio-sensitization and reduces chemo-resistance to temozolomide in GBM cell lines [37]. Cabozantinib is a potent, multitarget inhibitor of MET and VEGF receptor 2 [38]. NVP-BEZ235 (PI3K and mTOR a dual inhibitor) can inhibit the PI3K pathway to hinder glycolytic metabolism in GBM cells [39].

However, there were some limitations of our study. First, cuproptosis is a new concept, and there are few characteristic genes of cuproptosis, so it may affect the stability and applicability of the model on single-cell data. Second, the VEGF and CD99 signaling pathways were only detected by immunofluorescence, and further experiments are needed to prove their correlation with cuproptosis. Third, we found that EREG is closely related to PDL1 and FDX1, but further direct mechanisms are needed to reveal the relationship between them.

## Conclusion

**Fig. 14:**
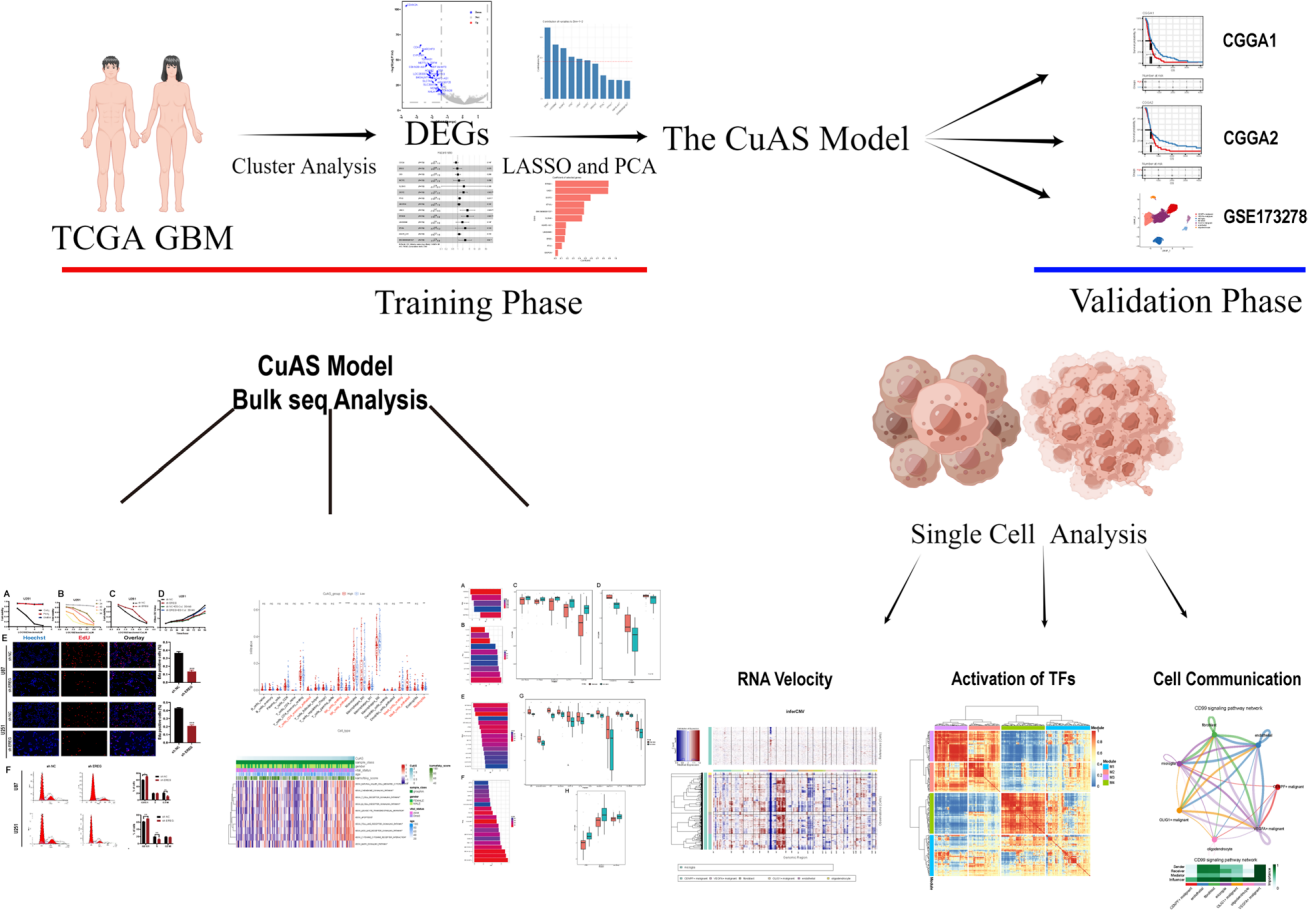
Graphical abstract of the CuAS model

Overall, we established a scoring model based on cuproptosis-related genes in glioblastoma samples (Fig. 14). High CuAS samples show more aggressive growth patterns and worse clinical outcomes than low CuAS samples. EREG, the core model gene, is an oncogenic gene that can affect immunity by influencing the expression of PDL1 and is closely related to cuproptosis by influencing the expression of FDX1. High CuAS GBM cells are found in VEGFA + malignant cells, and VEGF and CD99 is the differential pathway of specific cell communication between high and low CuAS groups. We assumed that targeting high CuAS samples may improve a patient’s prognosis. Moreover, novel potential compounds and immunotherapy can also be predicted. Taken together, CuAS can evaluate glioblastoma aggressiveness, modulate the cross-talk between VEGF/CD99 signaling, and provide support for immunotherapy and chemotherapy.

## Supplementary Information

**Additional file 1: Table S1.** Clinical characteristics of the three data sets. **Table S2.** shRNA sequence used in this study. **Table S3.** Primers sequence used in this study. **Table S4.** Antibodies used in this study.

**Additional file 2: Figure S1.** Batch Effect (A) and Batch Correction (B) for Single-Cell Data. **Figure S2.** Distribution of copper death score in single cell data. (A) Calculate CuAS based on the same method as before; (B) Calculate CuAS based on AUCell method; (C) Expression distribution of the detected characteristic genes in each cell type. **Figure S3.** Trajectory inference of tumor cells were depicted by UMAP. **Figure S4.** Based on the TF-target information of hTFtarget database, the activity of 16 TFS in each cell was calculated. **Figure S5.** Calculation of immune cell infiltration based on CIBERSORT and xCell method to observe the difference in cell infiltration between high and low CuAS groups. **Figure S6.** Prognostic efficacy of CuAS score on overall survival and progression-free survivain an immunotherapy cohort. (A) GSE131521. (B) phs001493. (C) IMvigor210. (D) PRJEB23709_ipiPD1. (E) NCT02684006. The unit of survival time is month. Patients were divided into groups based on CuAS score and maximum unified measurement.

## Data Availability

All data are available on public repositories, which are listed in the main context.

## Abbreviations

GBM: Glioblastoma multiforme
RNA-seq: RNA sequencing
TCGA: The cancer genome atlas cohort
CGGA: Chinese glioma genome atlas cohort
scRNA-seq: Single cell RNA-seq
qRT-PCR: Quantitative reverse transcription polymerase chain reaction
IHC: Immunohistochemistry
IF: Immunofluorescence
CCK-8: Cell counting kit-8
EdU: 5-Ethynyl-2’-deoxyuridine
EREG: Epiregulin
PCA: Principal component analysis
PD-L1: Programmed death-ligand 1 (PD-L1)
FDX1: Ferredoxin 1
VEGFA: Vascular endothelial growth factor A
OLIG1: Oligodendrocyte transcription factor 1
OS: Overall survival
RCD: Regulated cell death
SNV: Single nucleotide variant
CNA: Copy number variation
DEGs: Differentially expressed genes
HR: Hazard ratio
GSEA: Gene set enrichment analysis
ROC: Receiver operating characteristic
AUC: Area under the curve
DMEM: Dulbecco’s modified eagle’s medium
CSI: Connection specificity index
GDSC: Genomics of drug sensitivity in cancer
CCLE: Cancer cell line encyclopedia
CTRP: Cancer therapeutics response portal
NMF: Non-negative matrix factorization (NMF)
EGF: Epidermal growth factor
