# Supplementary material for: EREG is the core onco-immunological biomarker of cuproptosis and mediates the cross-talk between VEGF and CD99 signaling in glioblastoma
Source: J Transl Med. 2023 Jan 16;21:28. doi: 10.1186/s12967-023-03883-4 (PMC9843967; doi:10.1186/s12967-023-03883-4)
Supplement: Supplementary file 1 — Additional file 1: Table S1. Clinical characteristics of the three data sets. Table S2. shRNA sequence used in this study. Table S3. Primers sequence used in this study. Table S4. Antibodies used in this study. [file 12967_2023_3883_MOESM1_ESM.docx]

**Additional file 1: Table S1. Clinical characteristics of the three data sets**

**
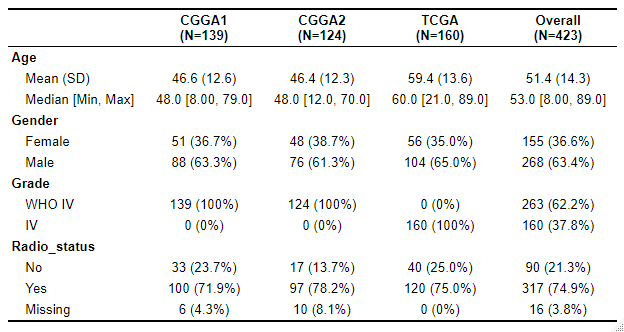
**

**Additional file 1: Table S2. shRNA sequence used in this study.**

| **Name** | **Sequence** | **Company** |
| --- | --- | --- |
| EREG-shRNA1 | GCTCATCATTTCACAGCTAAG | genecreate |
| EREG-shRNA2 | GCATAATTCCCGGATCACTAC | genecreate |
| EREG-shRNA3 | GGCTGTAAATAACGTTGAATG | genecreate |

**Additional file 1: Table S3. Primers sequence used in this study.**

| **Primers used for quantitative RT-PCR** | | |
| --- | --- | --- |
| **Name** | **Forward-primer** | **Reverse-primer** |
| ITPRID1 | TCTGGTGATCGAGGACTTCC | ACCTCCTCAGTATCACACACT |
| UNCX | TGGCATCTGGTAACTTTTGACTG | TGGTAGACAACAGGTGCTGC |
| DCST2 | TGGTATTGCACGGACACCTA | TGTTTCACCAGTGGGCTTG |
| ETV3L | CGGCTTTTCAGAAAGTGAGG | CATCGTCAGAGTCGGCATGA |
| RP11-863P13.3 | AAGACTCCGACTCCAGCTCT | CTGACATTACTGCAGGCCCA |
| SLC6A3 | CCAGCCGCCAGGTGTGTATC | GGTTTCCCCGAGTTTCATAGGTG |
| AGAP2−AS1 | CGTCAGCCCAGCCTCTTATG | TCAGGCCTCTCTGGGTGAG |
| LINC00968 | CCTGGTCATACTGTGGGAAAC | GGTGGGTCTTGATATGTTTTGAC |
| EREG | ACTGTACCACCATCCACTCAAC | CTGTTCCGTCCCAGTAGAT |
| PTX3 | GTGACGTTGACATCCGTAAAGA | GCCGGACTCATCGTACTCC |
| GAPDH | AAAAGCATCACCCGGAGGAGAA | AAGGAAATGAATGGGCAGCCG |
| SBSPON | TGCGGGTGCTCGCTTCGGCAGC | CCAGTGCAGGGTCCGAGGT |

**Additional file 1: Table S4. Antibodies used in this study.**

| **Name** | **Company** | **Catalog Number** | **Assay** |
| --- | --- | --- | --- |
| FDX1 | Proteintech | #12592-1-AP | WB |
| PDL1 | Proteintech | #66248-1-Ig | WB |
| UNCX | Abmart | #PU318142 | IHC |
| SLC6A3 | Proteintech | #22524-1-AP | IHC |
| EREG | Abmart | #PK94578 | WB |
| PTX3 | Proteintech | #13797-1-AP | IHC |
| VEGFA | Proteintech | #66828-1-Ig | IF |
| CD99 | Proteintech | #A2028 | IF |
| GAPDH | Proteintech | #60004-1-Ig | WB |
| HRP-conjugated Affinipure Goat Anti-Mouse IgG(H+L) | Proteintech | #SA00001-1 | WB |
| HRP-conjugated Affinipure Goat Anti-Rabbit IgG(H+L) | Proteintech | # SA00001-2 | WB |
