# Supplementary material for: EREG is the core onco-immunological biomarker of cuproptosis and mediates the cross-talk between VEGF and CD99 signaling in glioblastoma
Source: J Transl Med. 2023 Jan 16;21:28. doi: 10.1186/s12967-023-03883-4 (PMC9843967; doi:10.1186/s12967-023-03883-4)

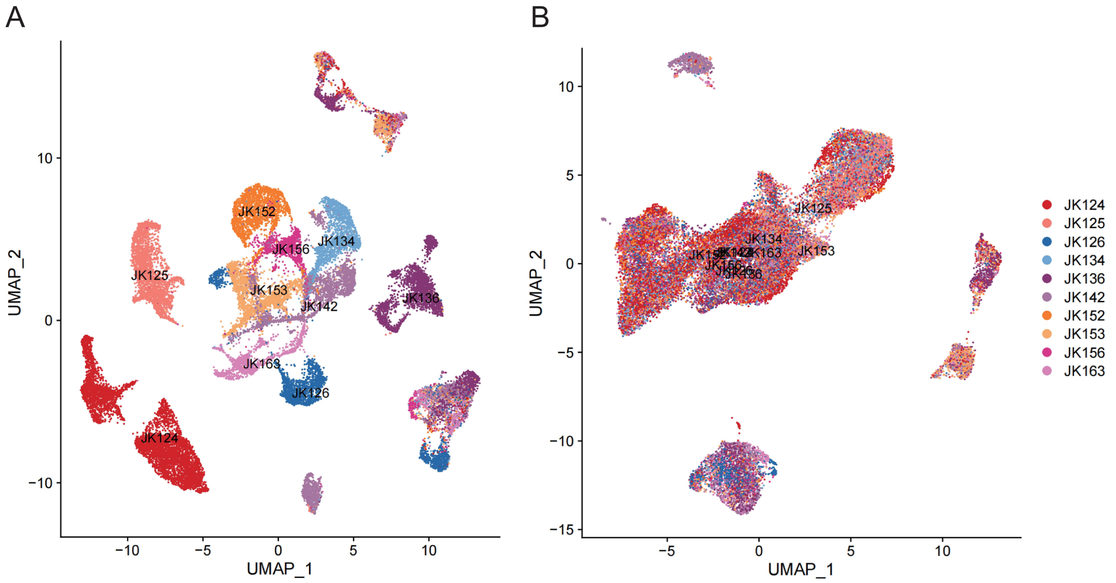

Figure S1. Batch Effect (A) and Batch Correction (B) for Single-Cell Data.

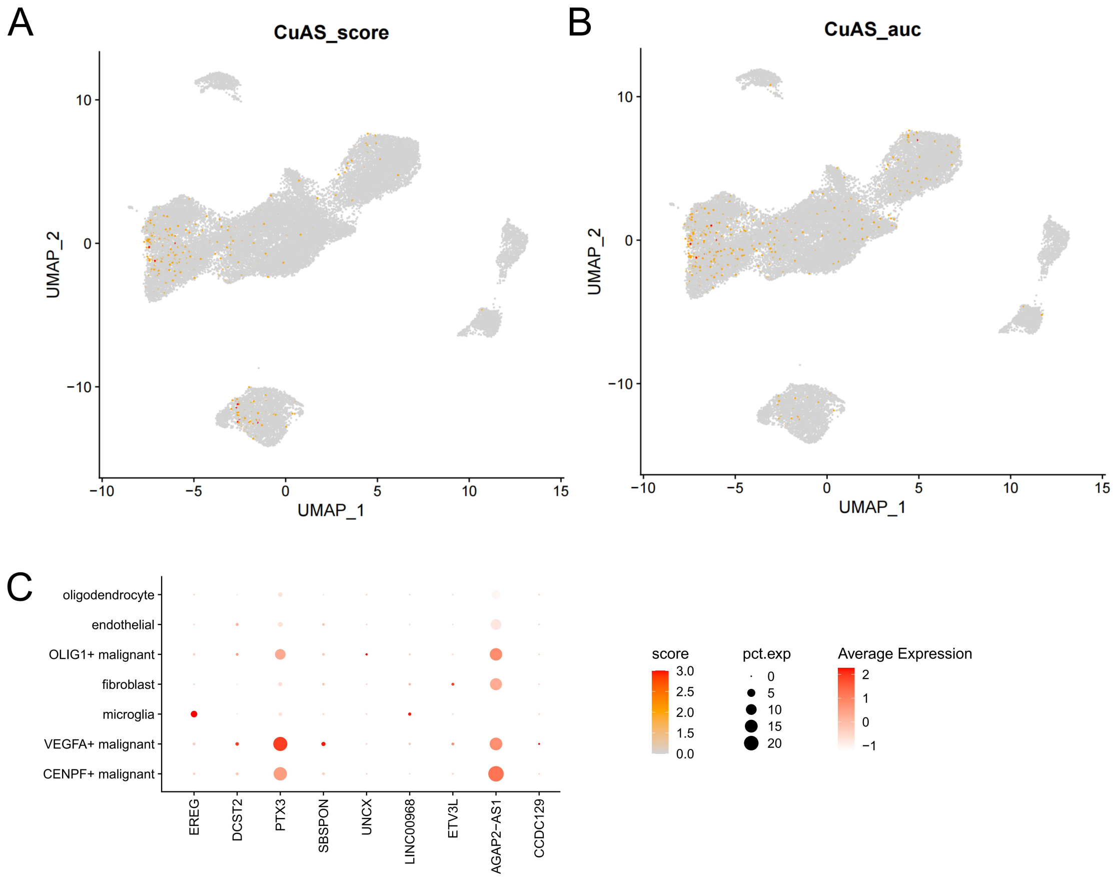

Figure S2. Distribution of copper death score in single cell data. (A) Calculate CuAS based on the same method as before; (B) Calculate CuAS based on AUCell method; (C) Expression distribution of the detected characteristic genes in each cell type.

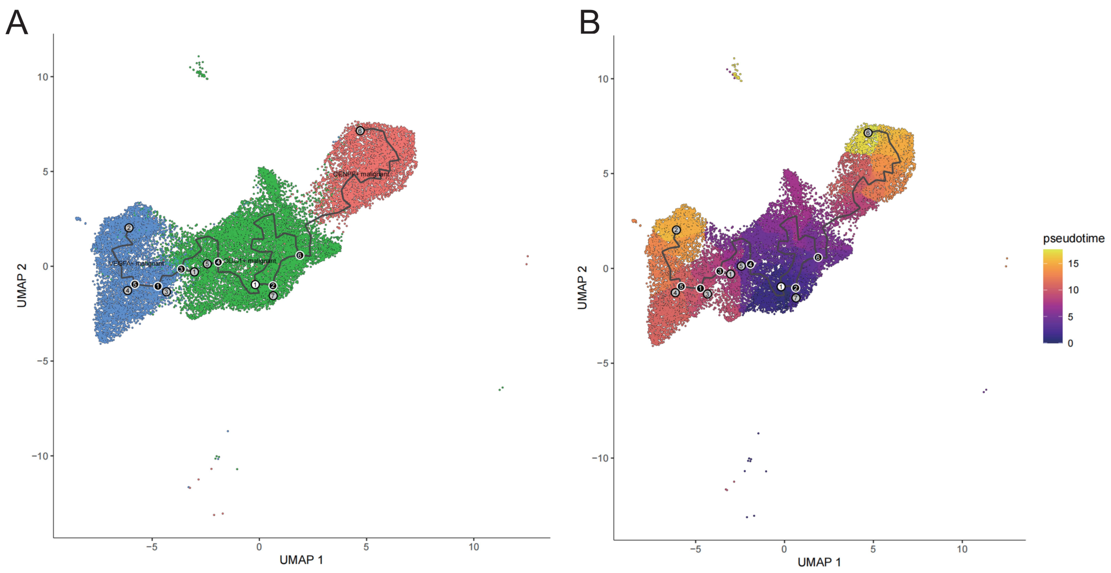

Figure S3. Trajectory inference of tumor cells were depicted by UMAP.

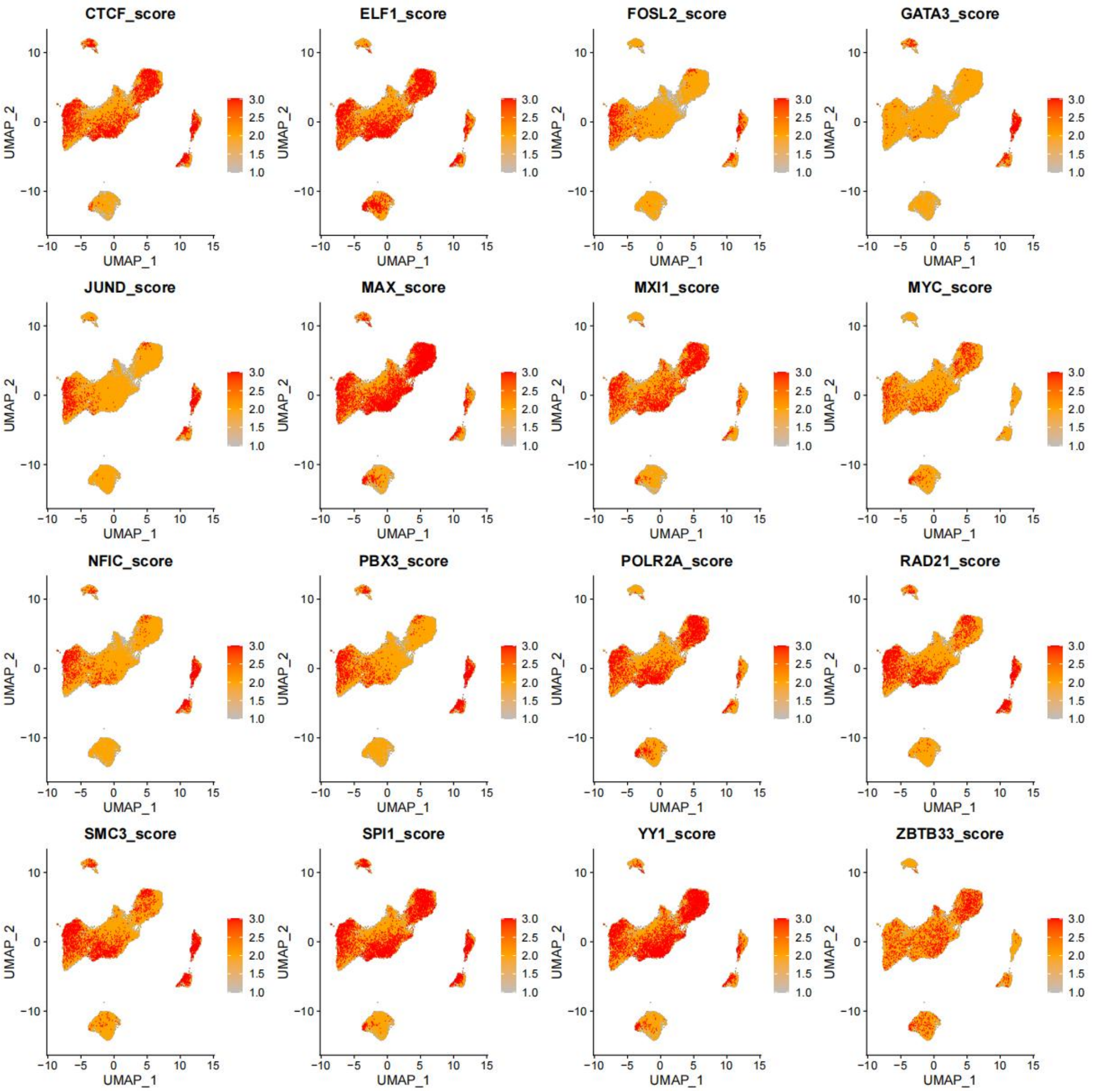

Figure S4. Based on the TF-target information of hTFtarget database, the activity of 16 TFS in each cell was calculated.

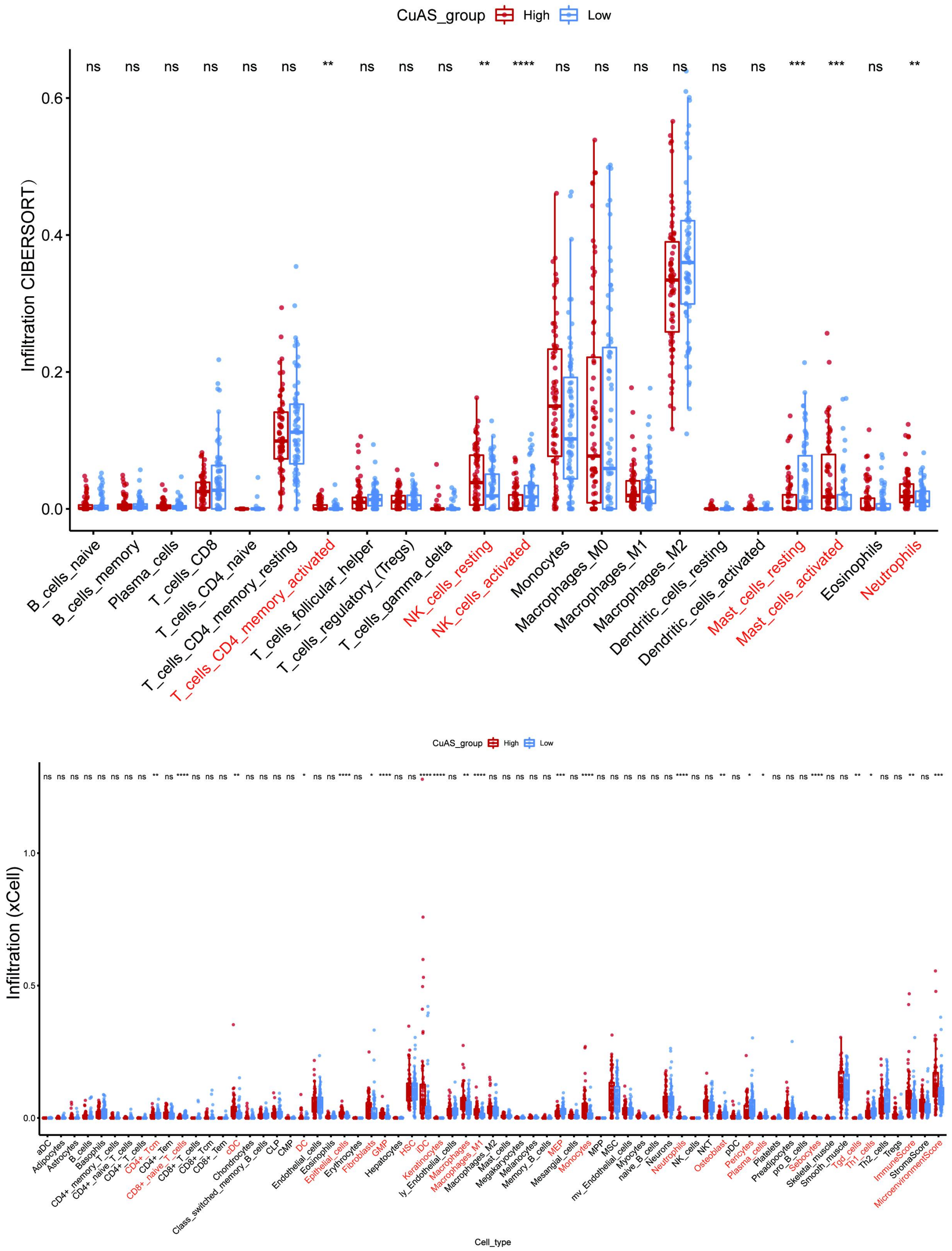

Figure S5. Calculation of immune cell infiltration based on CIBERSORT and xCell method to observe the difference in cell infiltration between high and low CuAS groups.

Figure S6.Prognostic efficacy of CuAS score on overall survival and progression-free survivain an immunotherapy cohort. (A) GSE131521. (B) phs001493. (C) IMvigor210. (D) PRJEB23709_ipiPD1. (E) NCT02684006. The unit of survival time is month. Patients were divided into groups based on CuAS score and maximum unified measurement.

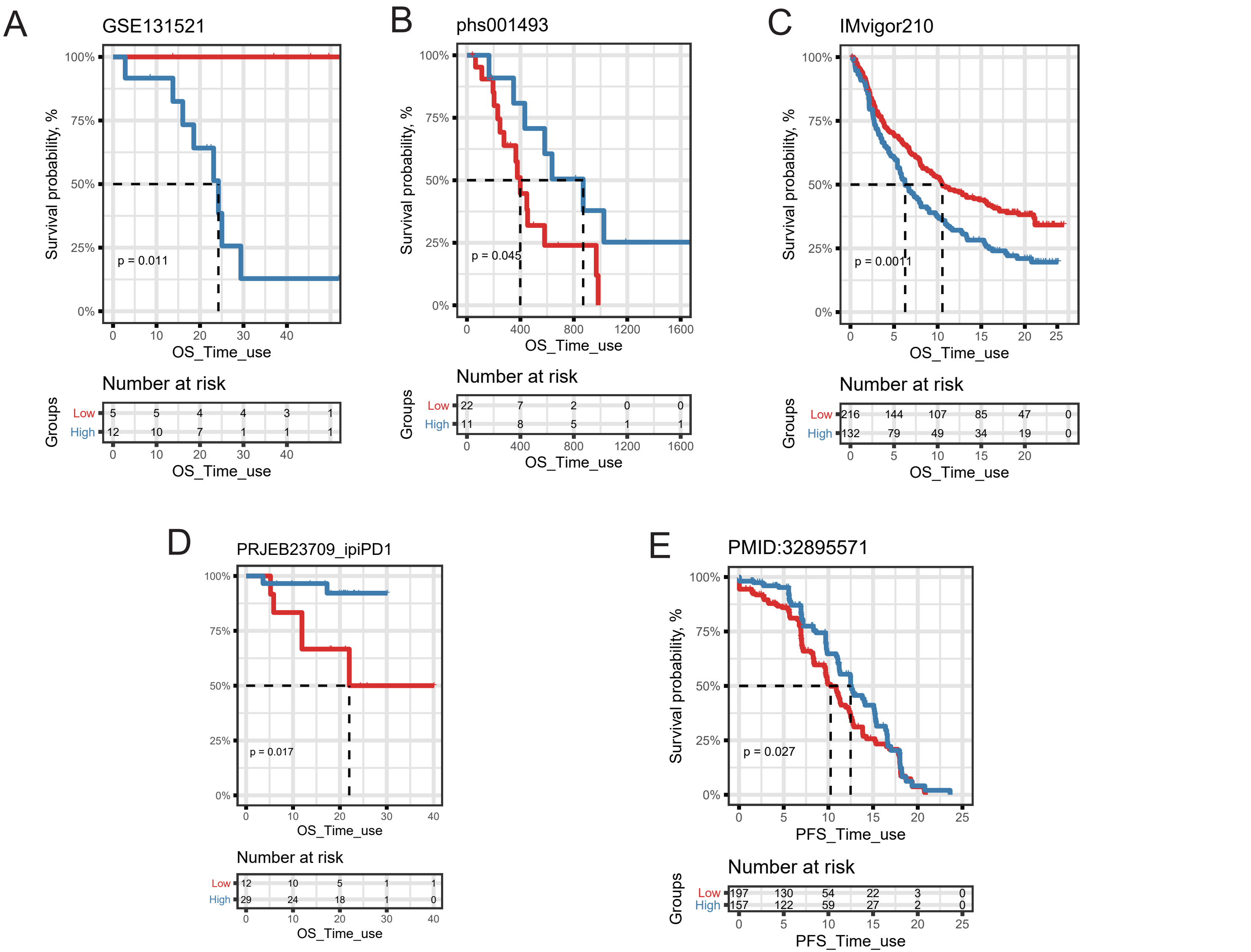

Supplement: Supplementary file 2 — Additional file 2: Figure S1. Batch Effect (A) and Batch Correction (B) for Single-Cell Data. Figure S2. Distribution of copper death score in single cell data. (A) Calculate CuAS based on the same method as before; (B) Calculate CuAS based on AUCell method; (C) Expression distribution of the detected characteristic genes in each cell type. Figure S3. Trajectory inference of tumor cells were depicted by UMAP. Figure S4. Based on the TF-target information of hTFtarget database, the activity of 16 TFS in each cell was calculated. Figure S5. Calculation of immune cell infiltration based on CIBERSORT and xCell method to observe the difference in cell infiltration between high and low CuAS groups. Figure S6. Prognostic efficacy of CuAS score on overall survival and progression-free survivain an immunotherapy cohort. (A) GSE131521. (B) phs001493. (C) IMvigor210. (D) PRJEB23709_ipiPD1. (E) NCT02684006. The unit of survival time is month. Patients were divided into groups based on CuAS score and maximum unified measurement. [file 12967_2023_3883_MOESM2_ESM.pdf]
